# Structural basis for specific DNA sequence recognition by the transcription factor NFIL3

**DOI:** 10.1016/j.jbc.2024.105776

**Published:** 2024-02-19

**Authors:** Sizhuo Chen, Ming Lei, Ke Liu, Jinrong Min

**Affiliations:** Hubei Key Laboratory of Genetic Regulation and Integrative Biology, School of Life Sciences, Central China Normal University, Wuhan, China

**Keywords:** NFIL3, bZIP domain, C/EBP-related protein, disease-associated mutants, DNA crystal structure

## Abstract

The CCAAT/enhancer-binding proteins (C/EBPs) constitute a family of pivotal transcription factors involved in tissue development, cellular function, proliferation, and differentiation. NFIL3, as one of them, plays an important role in regulating immune cell differentiation, circadian clock system, and neural regeneration, yet its specific DNA recognition mechanism remains enigmatic. In this study, we showed by the ITC binding experiments that NFIL3 prefers to bind to the TTACGTAA DNA motif. Our structural studies revealed that the α-helical NFIL3 bZIP domain dimerizes through its leucine zipper region, and binds to DNA via its basic region. The two basic regions of the NFIL3 bZIP dimer were pushed apart upon binding to DNA, facilitating the snug accommodation of the two basic regions within the major grooves of the DNA. Remarkably, our binding and structural data also revealed that both NFIL3 and C/EBPα/β demonstrate a shared preference for the TTACGTAA sequence. Furthermore, our study revealed that disease-associated mutations within the NFIL3 bZIP domain result in either reduction or complete disruption of its DNA binding ability. These discoveries not only provide valuable insights into the DNA binding mechanisms of NFIL3 but also elucidate the causal role of NFIL3 mutations in disease pathogenesis.

The CCAAT/enhancer-binding protein (C/EBP) transcription factors belong to the basic region-leucine zipper (bZIP) family of transcription factors, which play crucial roles in tissue development, cell proliferation, and differentiation (1, 2, 3, 4, 5, 6). In 1988, the first C/EBP transcription factor was discovered and cloned (7). It was termed the CCAAT/enhancer binding protein due to its ability to bind to the CCAAT box within a few promoters and various viral enhancers (8). In humans, ten C/EBP transcription factors have been identified, including six C/EBP proteins (named C/EBPα-ζ), and four C/EBP-related proteins, TEF, HLF, DBP, and NFIL3 (8, 9, 10, 11). All of these proteins are characterized by the presence of a C-terminal bZIP domain, except NFIL3, which possesses a bZIP domain at its N-terminus (Fig. 1*A*).

**Figure 1:**
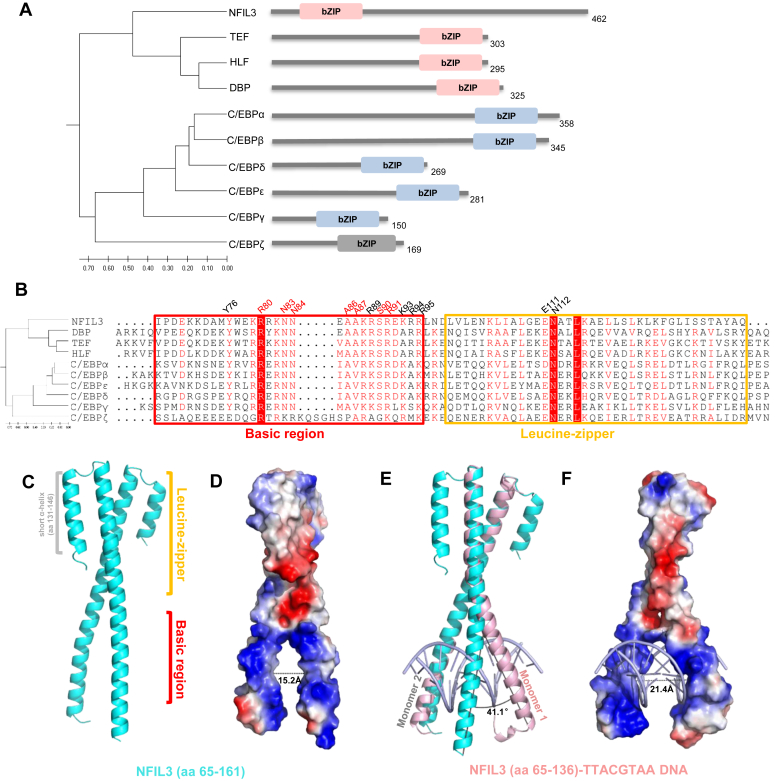
**Overall structures of the NFIL3 bZIP domain in apo form and complex with the TTACGTAA DNA.***A*, domain organization of the human C/EBP family proteins. NFIL3 (UniProt: Q16649); TEF (UniProt: Q10587); HLF (UniProt: Q16534); DBP (UniProt: Q10586); C/EBPα (UniProt: P49715); C/EBPβ (UniProt: P17676); C/EBPδ (UniProt: P49716); C/EBPε (UniProt: Q15744); C/EBPγ (UniProt: P53567); C/EBPζ (UniProt: P35638). bZIP: basic region-leucine zipper domain. The phylogenetic tree was generated using the amino acid sequences of the bZIP domain by MEGA11 (63). NFIL3, TEF, HLF, and DBP constitute the first subfamily colored in *pink*, C/EBPα, C/EBPβ, C/EBPδ, C/EBPε, and C/EBPγ form the second subfamily colored in *blue*, and C/EBPζ alone is shown here as a subfamily (*gray*). The numbers below the phylogenetic tree represent evolutionary branch lengths. *B*, sequence alignment of the bZIP domain of human C/EBP family of proteins. The basic region and leucine zipper are boxed with a *red box* and a *yellow box*, respectively. The base and backbone interacting residues in the basic region of NFIL3 are numbered in *red* and *black* above the sequence, respectively. The residues in the leucine zipper that affect the DNA binding are numbered in *black* above the sequence. *C*, overall structure of the NFIL3 bZIP domain (aa 65–161) in apo form. The extra short α-helix (aa 131–146) is colored in *gray*. The basic region and leucine zipper region are colored in *red* and *yellow*, respectively. *D*, the electrostatic surface representation of the NFIL3 bZIP domain (aa 65–161). The distance for the widest point in the NFIL3 bZIP domain is labeled. *E*, structural alignment of the NFIL3 bZIP domain in apo form and the NFIL3-TTACGTAA DNA complex. The labeled angle represents the widened angle between two basic regions in the NFIL3-DNA complex compared to the NFIL3 bZIP domain apo state. *F*, the electrostatic surface representation of NFIL3 (aa 65–136) in the DNA complex. The number indicates the distance between two basic regions at the widest point in the NFIL3-TTACGTAA DNA complex.

The bZIP domain consists of a basic region (BR) and a leucine zipper region with a sequence of heptad repeats (1, 12). The leucine zipper region is responsible for the dimerization of the bZIP domain, allowing the bZIP proteins to form either homodimers or heterodimers. The dimerization is essential for them to bind to specific target DNA sequences (13). Based on the sequence alignment of the bZIP domain, the C/EBP family of proteins could be divided into three subfamilies (Fig. 1*A*), each reported to exhibit a preferred DNA binding motif. Within each C/EBP protein subfamily, the sequence similarity of the basic region is greater than 72% (Fig. 1*B*), suggesting that proteins within the same subfamily share a common mechanism in recognizing DNA sequences. By analyzing the reported DNA binding sequences of the C/EBP proteins, it is revealed that these C/EBP proteins recognize a consensus sequence of TT(N4)AA (14, 15, 16, 17, 18, 19, 20). Among the ten human C/EBP transcription factors, C/EBPα and C/EBPβ have been extensively studied to play critical roles in the inflammatory response, especially in macrophage activation and rapid granulopoiesis following the stimulation by cytokines, and control the divergence of the common dendritic cell progenitor (CDP) into either conventional type 1 dendritic cells (cDC1) or conventional type 2 dendritic cells (cDC2) by competing with nuclear factor interleukin-3 regulated protein (NFIL3) at the −165 kb Zeb2 enhancer (21, 22, 23, 24).

NFIL3, also known as E4BP4, was originally found to repress transcription (25). NFIL3 plays a vital role in regulating immune cell differentiation and nerve regeneration (26, 27, 28). It is also a key component of the circadian clock system and functions as a negative regulator, exerting inhibitory control over the circadian oscillation of PER2 in the cell-autonomous core clock (29). Additionally, NFIL3 competes with DBP, HLF, and TEF for binding to the same consensus DNA binding site (TTACGTAA motif) during circadian regulation to mediate circadian repression (20, 29, 30, 31). Now we know that NFIL3 acts as not only a transcriptional repressor but also a trans activator of *IL-3* gene expression in T cells by recognizing the same consensus TTACGTAA DNA binding site (16, 19, 32, 33). In Baf-3 and FL5.12 cells, NFIL3 is a delayed early transcription factor that is induced by *IL-3* stimulation. When expression of NFIL3 is upregulated in FL5.12 cells, it stimulates the delay of apoptosis caused by deprivation of *IL-3* without promoting cell division, which suggests that the induction of NFIL3 is one of the mechanisms by which *IL-3* suppresses apoptosis (19).

To date, among the C/EBP family of proteins, only complex structures of the bZIP domains of C/EBPα and C/EBPβ bound to the TTGCGCAA DNA sequence have been determined (34, 35, 36). However, the DNA recognition mechanism of NFIL3, another critical member of the C/EBP family, remains unclear. Here, our ITC results showed that NFIL3 prefers a TTACGTAA motif, so we determined the crystal structures of the NFIL3 bZIP domain in its apo form and bound to DNA. We then examined the DNA binding specificities of the C/EBP protein family of proteins and found that both C/EBPα and C/EBPβ also show a preference towards recognizing the TTACGTAA DNA motif. This finding was further confirmed by our structures of C/EBPα and C/EBPβ in complex with the TTACGTAA DNA. We also investigated the DNA binding affinity and transcriptional activation ability of disease-associated mutants of NFIL3 using isothermal titration calorimetry (ITC) and luciferase reporter assays, and found that the disease-associated mutants decrease or disrupt the DNA-binding ability of NFIL3, which in turn reduced the transcriptional activity.

## Results

### bZIP domain of NFIL3 specifically binds to the TTACGTAA DNA motif

To quantitatively explore the DNA binding sequence specificity of NFIL3, we conducted isothermal titration calorimetry (ITC) binding assays using a palindromic sequence of CATTACGTAATG derived from the previously reported consensus sequences (19, 20). Our ITC results showed that the bZIP domain of NFIL3 displayed a binding affinity to the CATTACGTAATG DNA with a *K*_d_ of ∼0.042 μM (Table 1 and Fig. S1). When we mutated a single or more nucleotide on the first TTAC repeat of the palindromic CATTACGTAATG DNA sequence, the mutant sequences displayed varying degrees of reduced binding affinities to NFIL3 (Table 1 and Fig. S1). We also mutated the CATTACGTAATG sequence to CgTTACGTAATG to check if the sequence surrounding the consensus DNA motif affected the binding affinity. Our ITC results showed that this mutation had just a marginal effect on the NFIL3 binding (*K*_d_ = 0.067 μM compared to 0.042 μM), indicating that the flanking DNA sequence did not affect its binding to NFIL3 (Table 1 and Fig. S1).

**Table 1:** Binding affinities of the NFIL3 bZIP (aa 65–161) protein to different DNA sequences

DNA sequences	*K*_d_ (μM)
5′-CA**TTACGTAA**TG-3′^a^ 3′-GT**AATGCATT**AC-5′	0.042 ± 0.007
5′-CA**cTACGTAA**TG-3′ 3′-GT**gATGCATT**AC-5′	2.43 ± 0.15
5′-CA**TcACGTAA**TG-3′ 3′-GT**AgTGCATT**AC-5′	1.66 ± 0.11
5′-CA**TTgCGTAA**TG-3′ 3′-GT**AAcGCATT**AC-5′	0.18 ± 0.02
5′-CA**TTAtGTAA**TG-3′ 3′-GT**AATaCATT**AC-5′	0.52 ± 0.03
5′-CA**ccgCGTAA**TG-3′ 3′-GT**ggcGCATT**AC-5′	6.89 ± 0.34
5′-CA**ccgCGcgg**TG-3′ 3′-GT**ggcGCgcc**AC-5′	WB
5′-CA**ccgtacgg**TG-3′ 3′-GT**ggcatgcc**AC-5′	WB
5′-Cg**TTACGTAA**TG-3′ 3′-Gc**AATGCATT**AC-5′	0.067 ± 0.014
5′-CA**TTAmcGTAA**TG-3′ 3′-GT**AATGmcATT**AC-5′	0.59 ± 0.08
5′-CA**TTAtGTAA**TG-3′ 3′-GT**AATGCATT**AC-5′ mismatch	0.29 ± 0.03

Abbreviation: WB, weak binding.

a DNA sequence used for crystallization in this study. Protein recognition sequences are bolded. The mutated nucleotides are shown in lower case letters.

### Overall structures of the bZIP domain of NFIL3 and its complex with the TTACGTAA DNA

The recognition of the TTACGTAA sequence motif in gene promoters by NFIL3 has been established (19, 20), but the specific recognition mechanism remains unclear. To address this, we first determined the structure of the bZIP domain of NFIL3 (aa 65–161) in its apo form. The construct (aa 65–161) of the NFIL3 bZIP domain contained an extra short α-helix (aa 131–146), in addition to the canonical bZIP α-helix domain (aa 73–126). The short α-helix is almost antiparallel to the long bZIP α-helix (Fig. 1*C*). Unfortunately, we were not able to crystallize this construct with DNA. Consequently, we designed some shorter constructs in an attempt to obtain DNA complex crystals, which ultimately led to the successful crystallization and structure determination of the NFIL3-DNA complex using a shorter NFIL3 construct (aa 65–136).

Similar to other bZIP domains, NFIL3 formed a dimer in both apo and complex structures, and each NFIL3 monomer consisted of a DNA-binding basic region (BR) and a leucine zipper coiled-coil motif (LZ) for dimerization (Fig. 1, *C* and *E*). The leucine zipper region is rich in hydrophobic residues, facilitating dimerization through hydrophobic interactions (Fig. S2*C*), which was also confirmed by our gel filtration chromatography results (Fig. S2, *A* and *B*). The basic region is rich in basic residues, and the two basic regions were pulled together by the leucine zipper, forming a pliers-like structure, which provided a positively charged surface in its two handles to hold the negatively charged phosphate backbone of DNA (Figs. 1, *D* and *F* and S2, *D* and *E*). As expected, the short α-helix at the C-terminus of the long construct (aa 65–161) in the apo structure was away from the DNA-binding site, and would not provide any interactions with DNA (Fig. 1, *C* and *E*). Our ITC results with the short construct (aa 65–136) showed that it exhibited a comparable binding affinity to the longer construct (Fig. S3). Structural comparison of the apo and DNA complex structures of the NFIL3 bZIP domain revealed that, while the leucine zipper region did not undergo any change, the two α-helical basic regions from the dimer were squeezed out when bound to the DNA duplex, suggesting that the DNA binding caused the structural change of the α-helical basic regions of NFIL3 bZIP. The angle between the two α-helical basic regions was widened as much as ∼41°, and the width between the two basic regions of the bZIP dimer was expanded from 15.2 Å to 21.4 Å when bound to DNA (Fig. 1, *D* and *F*).

### Structural basis for specific binding of the TTACGTAA sequence by NFIL3

**Figure 2:**
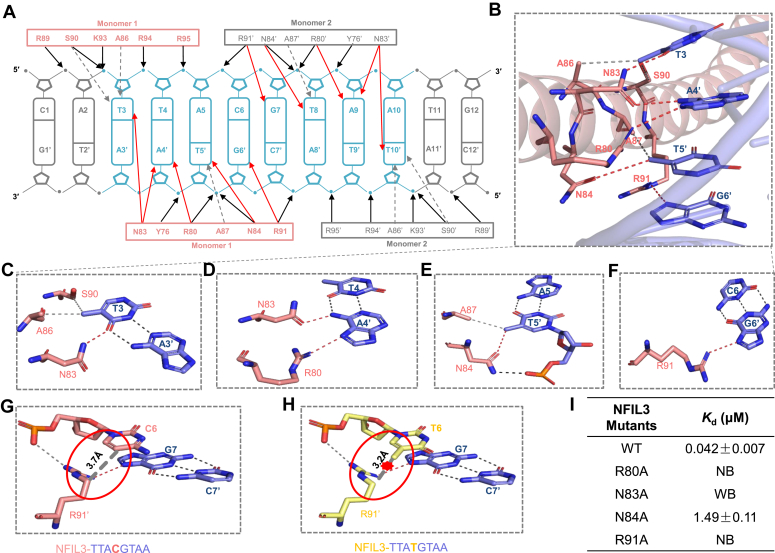
**Structural basis for specific binding of the TTACGTAA sequence by NFIL3.***A*, schematic diagram of the bZIP domain of NFIL3 bound to TTACGTAA DNA. Hydrogen bonds of DNA base-specific and backbone interactions are represented as *red* and *black solid arrows*, respectively. Van der Waals interactions between the residues and bases are represented as *gray dotted arrows*. *B*, the base-specific recognition by the NFIL3 bZIP domain in major grooves of DNA. Residues and DNA bases are shown in *sticks*, and colored *salmon* and *blue*, respectively. The interactions are shown in the same way as in (*A*). *C*, interactions between the T3/A3' base pair and the NFIL3 bZIP domain. *D*, interactions between the T4/A4' base pair and the NFIL3 bZIP domain. *E*, interactions between the A5/T5' base pair and the NFIL3 bZIP domain. *F*, interactions between the C6/G6' base pair and the NFIL3 bZIP domain. *G* and *H*, interactions between the C6 base (*G*) and T6 base (*H*) and the NFIL3 bZIP domain. The different bases are colored in *salmon* and *yellow*, respectively. R91' represents a residue on monomer2. Van der Waals interactions, backbone-mediated hydrogen bonds and base-specific interactions are represented in *gray*, *black* and *red*, respectively. In (*H*), the steric clash between the T6 base and R91' is highlighted by a *red star*. *I*, binding affinities of the NFIL3 (aa 65–161) mutants to the CATTACGTAATG DNA. NB, no detectable binding; WB, weak binding.

In the NFIL3-DNA complex, each NFIL3 monomer bound to half of the palindromic 5′-TTACGTAA-3′ recognition site, and the DNA binding modes of both monomers are virtually identical (Fig. 2*A*). For each monomer, seven residues, including R80, N83, N84, A86, A87, S90, and R91, were involved in direct base interactions with all four base pairs in the TTAC repeat (Fig. 2, *A* and *B*). In addition, Y76, R89, K93, R94, and R95 were exclusively involved in interacting with the phosphate backbone of the DNA (Fig. 2*A*). For brevity, we focused our discussion on one monomer.

For the first base pair T3/A3' of the TTAC repeat, the 5-position methyl group of T3 formed van der Waals interactions with the side chains of A86 and S90, while the O4 atom of the thymine formed a hydrogen bond with the side chain of N83 (Fig. 2*C*). Our ITC results showed that replacing T3 with C reduced its binding affinities by ∼58-fold (Table 1 and Fig. S1). Based on the structure of the NFIL3-TTACGTAA complex, we generated structural models of T3 DNA mutants in complex with NFIL3, and structural analysis revealed when T3 was mutated to either C, A, or G, the van der Waals interactions with A86 and S90 were disrupted (Fig. S4, *A*–*C*).

The second base pair T4/A4' formed two hydrogen bonds with NFIL3, one of which was formed between the N7 atom of A4' and the side chain of R80, and another between the exocyclic N6-amino group of A4' and N83 (Fig. 2*D*). Our ITC results showed that replacing T4 with C reduced its binding affinities by ∼39-fold (Table 1 and Fig. S1). When T4 was mutated to C, our structural modeling showed that the O6 atom of G4' or the O4 atom of T3 and the oxygen atom of the N83 carboxamide group were all hydrogen bond acceptors and could not form a hydrogen bond (Fig. S4, *D* and *E*). When T4 was mutated to A or G, T4' or C4' that is complementary with A4 or G4 either clashed or lost the hydrogen bond with the amino group of R80, and T4' also disrupted a hydrogen bond formed by N83 (Fig. S4, *F*–*H*).

The third base pair A5/T5' exhibited relatively weaker interactions with NFIL3. Its interactions primarily involved the 5-position methyl group of T5', engaging van der Waals interactions with A87 and forming a C-H•••O type hydrogen bond with N84 (35). N84 also formed a hydrogen bond with the phosphate backbone through the nitrogen atom of its carboxamide group (Fig. 2*E*). Our ITC results showed that replacing A5 with G resulted in a relatively weak reduction in binding affinity, ∼4-fold (Table 1 and Fig. S1). When A5 was mutated to G, C or T, both interactions were disrupted, interestingly, G5' or A5' could form new hydrogen bonds with R80 to offset the lost interactions from the mutation (Fig. S4, *I*–*K*).

In the fourth base pair, C6/G6', only the O6 atom in G6' formed a hydrogen bond with R91 (Fig. 2*F*). Our ITC results showed that replacing C6 with T reduced its binding affinities by ∼12-fold (Table 1 and Fig. S1). Previous study has shown that methylation of CpG sites in the promoter impaired NFIL3 recognition (37), but C/EBPβ displayed a stronger ability to DNA with cytosine methylation or a T/G mismatch (35, 36). Thus, to investigate whether cytosine methylation in CpG or T/G mismatch affects the NFIL3 recognition, we performed ITC assays, which showed that cytosine methylation or T/G mismatch of DNA exhibited a ∼14- or ∼7-fold reduction in binding affinity compared to the wild-type DNA (Table 1 and Fig. S1), consistent with our binding data for the C6 to T mutation DNA (Table 1 and Fig. S1). To explain how the C6 to T mutation affected its binding affinity to NFIL3, we determined the structure of NFIL3 in complex with the CATTATGTAACG DNA (Fig. 2*H*). In the NFIL3-CATTACGTAATG structure, the C5 atom of the CpG cytosine was 3.7 Å away from the C4 atom of R91' on monomer2. Substituting the central C with T led to the C7 atom in the T6 base forming a closer contact with R91', which was not favored (Fig. 2, *G* and *H*). Hence, our structure and ITC assays unequivocally demonstrated that the bZIP domain of NFIL3 exhibits a higher affinity towards the TTACGTAA DNA motif.

To validate the importance of the DNA sequence-specific binding residues of NFIL3, we mutated R80, N83, N84, or R91 to alanine individually, and examined their DNA binding ability by ITC assays. Our ITC results showed that mutating N84 of NFIL3 to alanine reduced its binding affinity by ∼35-fold compared to the wild-type NFIL3, while the R80A, N83A, and R91A mutants exhibited negligible or very weak binding (Figs. 2*I* and S5). To check if these mutants were properly folded, we utilized the differential scanning fluorimetry (DSF) assays to measure the melting temperatures of these mutants, and found that the melting temperatures of the mutants were largely unaltered, suggesting that mutations of these key amino acids did not significantly undermine the proteins’ stability (Fig. S6).

### Structural basis for preferential binding of the C/EBP family proteins to the TTACGTAA DNA sequence

To investigate the different preferential DNA binding sequences of the C/EBP subfamilies systematically, we performed ITC assays to measure the binding affinities of some representative C/EBP members, such as NFIL3, C/EBPα, C/EBPβ, and C/EBPζ, with reported preferential DNA sequences of each subfamily (14, 15, 16, 17, 18, 19, 20). Our ITC results showed that C/EBPα and C/EBPβ bound to the TTACGTAA DNA with a *K*_d_ of ∼0.354 μM and 0.018 μM, respectively, which were 1.4- or 3.8-fold stronger than to the TTGCGCAA DNA, *i.e.*, the reported consensus binding sequence of C/EBPα/β (Table 2 and Fig. S7). To understand why C/EBPα/β prefers recognizing the TTACGTAA DNA, we determined the structures of C/EBPα and C/EBPβ in complex with the TTACGTAA DNA, respectively (Fig. S8, *A* and *B*). Structural comparison of the C/EBPβ-TTACGTAA complex with the C/EBPβ-TTGCGCAA complex (PDB 1GU4) revealed that C/EBPβ formed two additional interactions involving the 5-position methyl group of T5' in the C/EBPβ-TTACGTAA structure, including van der Waals interactions with residue V285, and a C-H•••O hydrogen bond with residue N282 (Figs. S8*C* and S9*A*), which were also conserved in the C/EBPα-TTACGTAA structure (Fig. S9*B*). These interactions might contribute to the moderate increase in binding affinities to the TTACGTAA DNA sequence by C/EBPα and C/EBPβ.

**Table 2:** Binding affinities of the C/EBP family bZIP domain proteins to different DNA sequences

Protein	DNA sequences	*K*_d_ (μM）
NFIL3	5′-CA**TTACGTAA**TG-3′^a^ 3′-GT**AATGCATT**AC-5′	0.042 ± 0.007
	5′-CA**TTGCGCAA**TG-3′ 3′-GT**AACGCGTT**AC-5′	0.81 ± 0.07
C/EBPα	5′-CA**TTACGTAA**TG-3′^a^ 3′-GT**AATGCATT**AC-5′	0.35 ± 0.03
	5′-CA**TTGCGCAA**TG-3′ 3′-GT**AACGCGTT**AC-5′	0.48 ± 0.07
C/EBPβ	5′-CA**TTACGTAA**TG-3′^a^ 3′-GT**AATGCATT**AC-5′	0.018 ± 0.003
	5′-CA**TTGCGCAA**TG-3′ 3′-GT**AACGCGTT**AC-5′	0.069 ± 0.009
C/EBPζ	5′-CA**TTACGTAA**TG-3′^a^ 3′-GT**AATGCATT**AC-5′	NB
	5′-CA**TTGCGCAA**TG-3′ 3′-GT**AACGCGTT**AC-5′	NB
	5′-GA**TGATGTAA**TC-3′ 3′-CT**ACTACATT**AG-5′	NB

Abbreviation: NB, no detectable binding.

a DNA sequence used for crystallization in this study. Protein recognition sequences are bolded.

**Figure 3:**
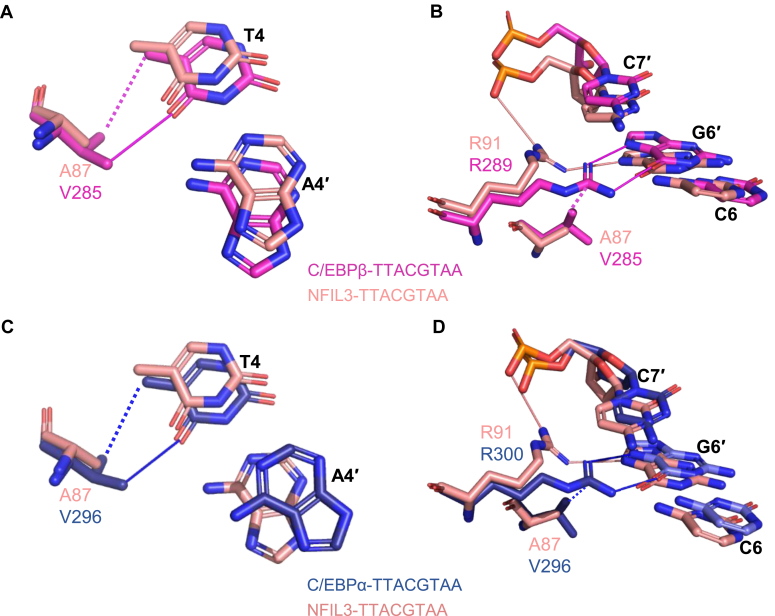
**Structural basis for preferential binding of the C/EBP family proteins to the TTACGTAA DNA.***A* and *B*, the interaction differences between NFIL3 and C/EBPβ bound to the TTACGTAA DNA. *C* and *D*, the interaction differences between NFIL3 and C/EBPα bound to the TTACGTAA DNA. Hydrogen bonds and Van der Waals interactions are represented as *solid* and *dotted lines*, respectively.

Our ITC results showed that the binding affinity of NFIL3 to the TTACGTAA DNA was ∼ 2-fold weaker than that of C/EBPβ (Table 2 and Fig. S7). Sequence analysis of the DNA binding basic region showed that the A87 in NFIL3 was substituted by V285 in C/EBPβ (Fig. 1*B*). By comparing the structures of the NFIL3-TTACGTAA complex and the C/EBPβ-TTACGTAA complex, we found that the longer side chain of V285 in C/EBPβ could form extra van der Waals interactions and an O•••H-C hydrogen bond with T4 on the T4/A4' base pair compared to A87 of NFIL3 (Fig. 3*A*). The longer side chain of V285 could also form van der Waals interactions with R289 to attract the latter to form bidentate hydrogen bonds with G6' in the C6/G6' base pair (Fig. 3*B*). All the extra interactions introduced by the V285 to A87 substitution in the C/EBPβ-DNA complex might result in a stronger binding of C/EBPβ to DNA than NFIL3.

**Figure 4:**
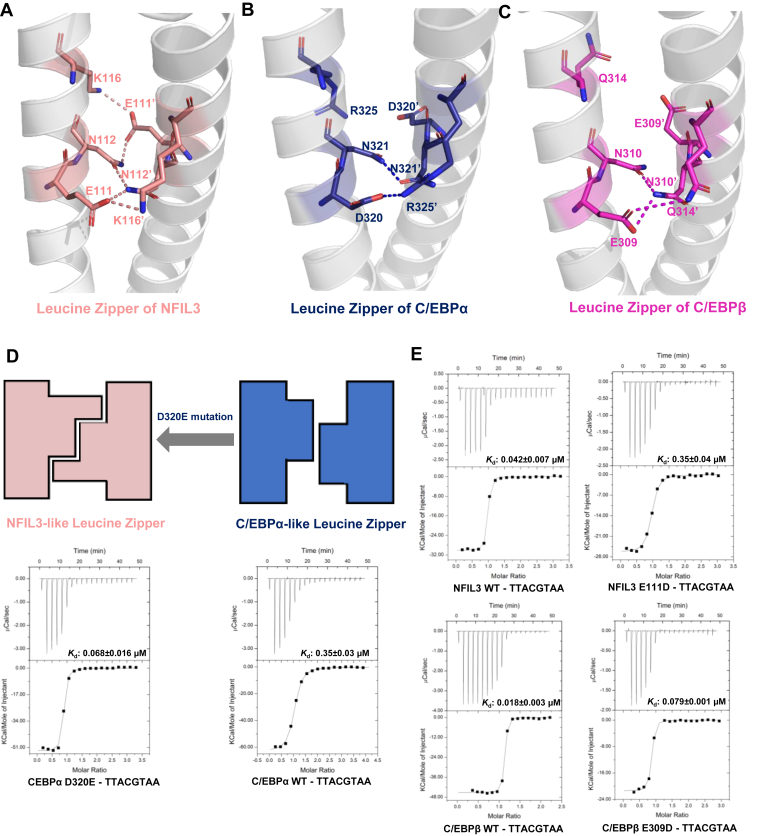
**Importance of the conserved DNA binding residues of leucine zipper region of the C/EBP family proteins.** Dimer interfaces of the leucine zipper region of NFIL3 (*A*), C/EBPα (*B*) and C/EBPβ (PDB 7UPZ) (*C*). The residues are shown in *sticks* and colored *salmon* (NFIL3), *blue* (C/EBPα), and *magenta* (C/EBPβ), respectively. *D*, a proposed model of the dimerization formation mediated by interfacial residues in the leucine zipper region. This model is further confirmed by ITC assays of the C/EBPα (aa 281–340) mutant binding to the TTACGTAA DNAs. *E*, binding affinities of the NFIL3 (aa 65–161) and C/EBPβ (aa 259–336) mutants to the TTACGTAA DNA.

Surprisingly, our binding results showed that C/EBPα bound to the TTACGTAA DNA of ∼8- to 20-fold weaker than NFIL3 and C/EBPβ (Table 2 and Fig. S7). Although C/EBPα exhibited a conserved binding mode to DNA in the basic region with C/EBPβ (Fig. 3, *C* and *D*), we found that C/EBPα carries an aspartic acid, D320, in the leucine zipper region, which is a glutamic acid in the corresponding position of the other C/EBP proteins (Fig. 1*B*). Structural comparison showed that the shorter side chain of D320 in C/EBPα prevented it from forming additional hydrogen bonds with N321' in the other monomer, unlike E111 in NFIL3 or E309 in C/EBPβ that formed a hydrogen bond with N112' in NFIL3 or N321' in C/EBPβ from the other monomer (Fig. 4, *A*–*C*). To further verify our observations, we carried out mutagenesis binding studies. Mutating D320 of C/EBPα to E enhanced its binding affinity by ∼5-fold compared to the wild-type C/EBPα (Fig. 4*D*). On the other hand, mutating E111 of NFIL3 or E309 of C/EBPβ to D resulted in ∼8- or 4-fold weaker binding affinities than their wild-type counterparts, respectively (Fig. 4*E*). When we mutated the E111-interacting residue N112 of NFIL3 to D, the dimerization of NFIL3 was not affected (Fig. S10*A*), but the binding affinity was also reduced 51-fold (Fig. S10*B*). These findings highlighted the importance of the conserved asparagine in DNA recognition.

Compared to other members of the C/EBP family, our ITC results showed that C/EBPζ exhibited no detectable binding to any tested DNA sequences in this study (Table 2 and Fig. S6), which was also corroborated by our electrophoretic mobility shift assays (EMSA) (Fig. S11*A*). We also explored the dimerization state of C/EBPζ by gel filtration chromatography and found that the recombinant protein of the C/EBPζ bZIP domain was still dimer (Fig. S11*B*). Further sequence alignment analysis revealed that the key DNA binding residues N83, N84, and R91 of NFIL3 are absent in C/EBPζ (Fig. 1*B*), potentially explaining the inability of C/EBPζ to bind to DNA.

### Effect of disease-associated mutations of the NFIL3 bZIP domain on its DNA binding ability

**Figure 5:**
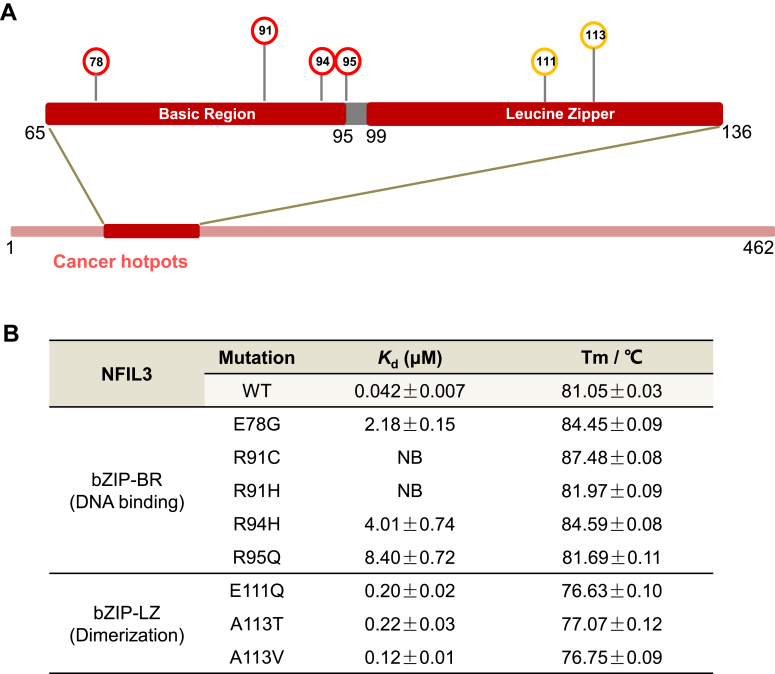
**Disease-associated mutations in the NFIL3 bZIP domain.***A*, the disease-associated mutations of the NFIL3 bZIP domain. Mutated amino acids are numbered. *B*, binding affinities of the CATTACGTAATG DNA to the NFIL3 (aa 65–161) disease-associated mutants. The determination of *K*_d_ values was repeated three times independently by ITC assays. Tm values for each sample were measured using DSF and calculated based on six independent experiments. NB, no detectable binding.

Based on the TCGA (The Cancer Genome Atlas) database (https://www.cancer.gov/tcga), the bZIP domain of NFIL3 harbors many hotspot disease mutations, including the E78G, R91C/H, R94H and R95Q mutations in the basic region, and the E111Q, A113T and A113V mutations in the leucine zipper (Fig. 5*A*). These mutations have been reported in different disease types in the TCGA database, including stomach adenocarcinoma (STAD), colon adenocarcinoma (COAD), and uterine corpus endometrial carcinoma (UCEC) (Fig. S12), but none of these mutations have been functionally characterized in terms of their DNA binding ability.

To explore how these mutations in the bZIP domain of NFIL3 affect its DNA binding ability and understand the pathogenesis of disease-related mutations of NFIL3, we made these disease-associated mutants of NFIL3 and measured their DNA binding ability by ITC. Our ITC results showed that the E78G, R91C/H, R94H, and R95Q mutations in the basic region of NFIL3 exhibited significantly reduced or no detectable DNA binding ability (Figs. 5*B* and S13). Our complex structure revealed that the mutations of E78G, R91C/H, R94H, and R95Q mainly reduced its binding to the DNA phosphate backbone (Fig. S14*A*). Among the mutations in the leucine zipper region, the E111Q, A113T, and A113V mutants moderately reduced the DNA binding affinity by ∼3- to 5-fold compared to the wild-type NFIL3 (Figs. 5*B* and S13). Structural analysis showed that although the mutated residues in the leucine zipper region did not directly interact with DNA, they affected the hydrophobicity of the hydrophobic region, which is critical for the dimerization of NFIL3 (Fig. S14*A*). Indeed, we found that the E111Q, A113T, and A113V mutations resulted in poor protein stability of NFIL3 compared to the wild-type NFIL3 (Fig. S14*B*). Collectively, disease-associated mutations in NFIL3 either directly or indirectly resulted in a decrease or disruption of its DNA-binding ability, potentially contributing to the pathogenesis of NFIL3-related diseases.

### Transcriptional activity of NFIL3 requires a fully functional DNA binding domain

**Figure 6:**
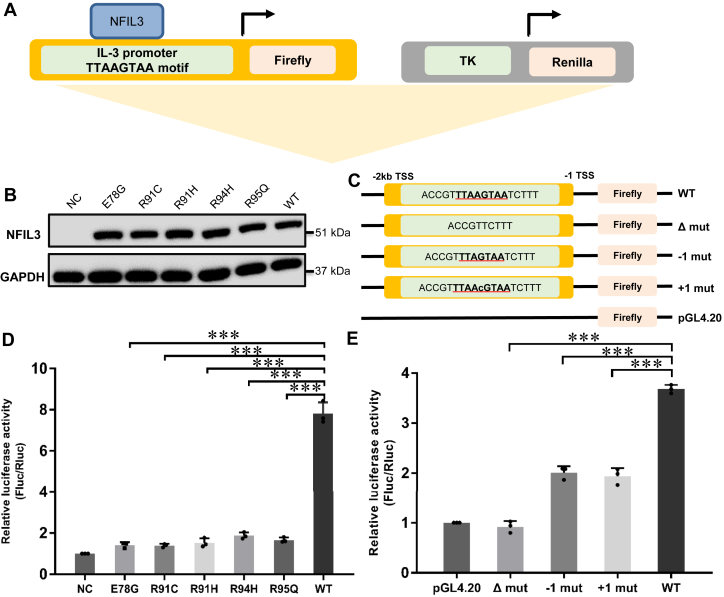
**Luciferase activity analysis of the NFIL3 disease-associated mutants.***A*, schematic representation of the dual-luciferase reporter system used to verify the activation of the *IL-3*-promoter by NFIL3. TK: promoter of Prl-TK vector. *B*, Western blot analysis of NFIL3 wild-type and mutants in HEK293T after 24 h transfection. *C*, schematic representation of the *IL-3*-promoter reporter assay. WT: normal *IL-3*-promoter containing TTAAGTAA region; Δ Mut: *IL-3*-promoter without TTAAGTAA motif; −1 or +1 Mut: *IL-3*-promoter TTAAGTAA motif mutants added or subtracted one central base in the central nucleotide AG motif; pGL4.20: empty vector as negative control. *D*, dual luciferase assays of wild-type NFIL3 and its mutants binding to WT *IL-3*-promoter. NC: pcDNA3.1 empty vector as negative control. *E*, dual luciferase assays of NFIL3 binding to WT and mutated *IL-3*-promoters in HEK293T. pGL4.20: empty vector as a negative control. Error bars represent SD from three replicates (n = 3) (∗∗∗*p* < 0.001).

Given ITC assays have shown that disease-associated mutations in the basic region of NFIL3 had a severe effect on the DNA binding, we further explored whether disease-associated mutations of NFIL3 affect its ability to activate transcription by luciferase reporter assays using a pGL4.20-*IL-3*-promoter plasmid with the NFIL3 binding site (TTAAGTAA) derived from JASPAR database (Fig. 6*A*). Compared with the TTACGTAA DNA motif, our ITC data showed that the NFIL3 bound to TTAAGTAA DNA with a *K*_d_ of 0.12 μM, albeit 3-fold weaker than the TTACGTAA DNA (Fig. S15*A*). Correspondingly, the luciferase reporter assay results showed that the TTAAGTAA DNA sequence exhibited slightly weaker transcriptional activity than that of the TTACGTAA DNA (Fig. S15*B*).

We first assessed the protein expression levels of the wild-type NFIL3 and its mutants, which showed that all of the NFIL3 mutants displayed similar expression levels to that of the wild-type NFIL3 (Fig. 6*B*). Subsequent luciferase reporter results revealed that all the NFIL3 disease variants displayed ∼4-fold reduction in luciferase activity compared to the wild-type NFIL3 (Fig. 6*D*), which was consistent with our findings from the ITC binding assays (Figs. 5*B* and S13).

To determine the importance of the DNA binding sequence (TTAAGTAA) of NFIL3, we constructed the pGL4.20-*IL-3*-promoter plasmids with different mutated NFIL3 binding sites and performed luciferase reporter assays (Fig. 6*C*). Our luciferase reporter assay showed that the deletion of the NFIL3 binding site (TTAAGTAA) resulted in a complete loss of transcriptional activation (Fig. 6*E*), strongly suggesting that the TTAAGTAA motif is the authentic binding site of the *IL-3* promoter by NFIL3. Furthermore, either increasing or decreasing nucleotide in between the TTA and TAA sequence of the TTAAGTAA sequence yielded a ∼2-fold reduction in luciferase activity (Fig. 6*E*), demonstrating the criticality of the integrity of the TTAAGTAA sequence for NFIL3 protein binding. Consequently, our luciferase reporter assays demonstrated that reduced transcriptional activity due to the decrease or disruption of the DNA binding ability of NFIL3 might be the main cause of NFIL3 mutation-mediated diseases in the bZIP domain.

## Discussion

NFIL3, as a transcription factor, actively participates in processes such as cell survival, metabolic regulation, and differentiation of immune cells and the circadian rhythm of organisms by effectively modulating the transcriptional levels of target genes (24, 38, 39, 40). Despite an increasing number of reports on the role of NFIL3, the molecular mechanisms by which NFIL3 selectively binds to specific promoters and regulates the expression of these target genes, as well as how its mutants contribute to disease initiation, remain unresolved.

In this study, we have discovered that the NFIL3, C/EBPα, and C/EBPβ, exhibited a binding preference for the TTACGTAA DNA sequence (Table 2 and Fig. S6). This observation is consistent with a recent study showing that C/EBPα/β binds to a TTACGTAA sequence within the Zeb2 enhancer. This creates a competitive context with NFIL3 for occupancy at this site, ultimately regulating the divergence of common dendritic progenitors (CDP) into either conventional type 1 dendritic cells (cDC1) or conventional type 2 dendritic cells (cDC2) (24). Additionally, we determined the structures of the NFIL3 bZIP domain alone and its complex with DNA and found that the width of the positively charged cavity formed by the basic regions of the bZIP dimer was widened when bound to DNA. Furthermore, we successfully determined the complex structures of C/EBPα and C/EBPβ bound to the TTACGTAA DNA, which allowed us to elucidate the molecular mechanism by which C/EBPα and C/EBPβ selectively recognize the TTACGTAA DNA. In conclusion, our study provides a mechanistic foundation for understanding how the C/EBP protein family selectively binds to specific promoters to regulate the expression of target genes.

While NFIL3 has been exclusively reported to function in transcriptional regulation as a homodimer, there is currently no documented evidence of NFIL3 operating as a heterodimer *in vivo*. Nonetheless, it has been reported that NFIL3 can form a heterodimer with CREB proteins *in vitro* (41, 42). Intriguingly, CREB binds to nearly identical DNA sequences as NFIL (43). We hypothesize that if the NFIL3-CREB heterodimer does exist *in vivo*, it might recognize similar sites as either the NFIL3 or CREB homodimer.

In recent years, there has been a growing trend toward utilizing regulated systems for gene transcription to modify genetic programs and related functional outcomes, including signaling and metabolic pathways (44, 45, 46). In our research, we revealed that the leucine zipper region in the bZIP domain not only plays a role in dimerization but also influences DNA binding ability. While previous studies have argued that transcription factors are considered 'undruggable' due to the perceived absence of pockets supporting specific small-molecule binding (47), attempts have been made to target the bZIP domains by covalently binding to cysteine residues in the basic region. However, this approach proves ineffective for bZIP domains lacking cysteines in their basic region (48). Our results suggested that modifying the leucine zipper region can have a comparable impact on regulating DNA binding ability, similar to the regulation observed in the basic region, raising a novel concept for developing drugs targeting the protein-protein interaction (PPI) interface of the leucine zipper region in homodimers to modulate DNA binding, as exemplified by our NFIL3 (N112D) mutant. This insight may be applicable to other members of the C/EBP family, such as C/EBPα and C/EBPβ, known for their involvement in various physiological activities, including synaptic plasticity, the etiology of glioblastoma, autophagy, and inflammation (49, 50, 51, 52).

## Experimental procedures

### Cloning, expression, and purification

The human NFIL3 (aa 65–136 and aa 65–161), C/EBPα (aa 281–340), C/EBPβ (aa 259–336) and C/EBPζ (aa 88–162) fragments were subcloned into the pET28-MKH8-SUMO vector to overexpress N-terminal His_6_ and Sumo-tagged fusion proteins, respectively. The recombinant plasmids were expressed using *Escherichia coli* BL21 (DE3) under the induction with 0.5 mM IPTG overnight at 14 °C. The cells were collected and resuspended in a lysis buffer of 20 mM Tris, pH 7.5, 500 mM NaCl, and 5% glycerol, which was then sonicated to break the cells at 4 °C. After sonication, the cells were centrifuged at 16,000*g* for 40 min, and then the collected supernatant of the target protein was purified using Ni-NTA resin (Qiagen). The recombinant protein was eluted and treated with Tobacco etch virus (TEV) to remove the His_6_ and Sumo-tag, the purified protein was further analyzed using affinity chromatography, anion-exchange column, and gel filtration column (GE Healthcare). The point mutations of NFIL3, C/EBPα, C/EBPβ, and C/EBPζ were introduced using mutated primers in PCR amplification and confirmed by sequencing, the mutant proteins were purified as mentioned for the wild-type protein. The final purified wild-type and mutant proteins were stored in a buffer containing 20 mM Tris, pH 7.5, and 150 mM NaCl. The primer information was summarized in Table S2.

### Size-exclusion chromatography analysis

The size-exclusion chromatogram assay was carried out to assess the oligomeric state of the NFIL3 bZIP domain protein and its mutants, as well as the binding state to DNA. The NFIL3 (2 mg/ml) was mixed with dsDNA in a molar ratio of 1:1.1 and incubated on ice for 30 min. The NFIL3 bZIP domain WT (2 mg/ml) apo, NFIL3 mutants, the NFIL3-DNA complex as well as the protein standard, were investigated using size-exclusion chromatography (Superdex 200 10/300 GL, GE) in a buffer of 20 mM Tris, pH 7.5 and 150 mM NaCl.

### Differential scanning fluorometry assay

The stability and DNA-binding potential of wild-type proteins and mutants were analyzed using differential scanning fluorometry assay. The protein samples were used at a final concentration of 1 mg/ml in a buffer with 20 mM Tris, pH 7.5, and 150 mM NaCl, and the fluorescent dye SYPRO Orange (Sigma) was used at a final concentration of 8×. For complex, each protein sample (1 mg/ml) was mixed with dsDNA in a molar ratio of 1:1.1 and incubated on ice for 30 min, and other components were the same as mentioned for apo samples. To measure the melting curves of apo protein and complex samples, the temperature was increased from 25 to 95 °C with a gradual rate of 2 °C/min using the Real-time PCR instrument (Light Cycler 480, Roche). The excitation wavelength was 465 nm and the emission wavelength was 580 nm. Melting curves were analyzed using GraphPad Prism 7, and Tm values for each sample were calculated based on six independent experiments.

### Isothermal titration calorimetry assay

The DNA oligonucleotides were synthesized by General Biosystems Co Ltd, and were dissolved in a buffer of 20 mM Tris, pH 7.5, and 150 mM NaCl. After adjusting the pH to around 7.5, the ssDNA was annealed to DNA duplex by PCR instrument. In the Isothermal titration calorimetry (ITC) assay, the final concentrations of wild-type NFIL3 (aa 65–161), C/EBPα (aa 281–340), C/EBPβ (aa 259–336) and their mutants were in the range of 15 to 80 μM and DNAs were in the range of 200 to 500 μM, respectively. The ITC assays were carried out by MicroCal PEAQ-ITC (Malvern) at 25 °C. Each titration consisted of 19 injections, and the *K*_d_ value was fitted using a one-site binding model by MicroCal ITC200 analysis software Origin 7.0 (Malvern). The ITC assays were conducted with three replicates (n = 3), and the errors represent the standard errors from the three replicates. NB: no detectable binding, indicating that there is no detectable heat during titration. WB: weak binding, indicating that the heat of the titration was too low to fit reliably.

### Electrophoretic mobility shift (EMSA) assay

The DNA oligonucleotides were synthesized by General Biosystems Co Ltd with the 5′ FAM-labeled and annealed to DNA duplex as mentioned for the ITC assay. TGATGTAA DNA sequence is GATGATGTAATC, TTACGTAA DNA sequence is CATTACGTAATG, TTGCGCAA DNA sequence is CATTGCGCAATG (only one strand is shown). For EMSA experiments, the FAM-labeled DNAs were diluted to a concentration of 10 μM by binding buffer containing 20 mM Tris, pH 7.5, and 150 mM NaCl, and incubated with proteins for 30 min at 4 °C at a final protein concentration of 6 μM, 12 μM, 24 μM, and 48 μM, respectively. Then, the reactions were resolved on 6% native acrylamide gels in 0.5× TBE buffer under an electric field of 100 V for about 2.5 h on ice. The gels were visualized on Fluor Chem R (Li-Cor, GelView 6000Pro Ⅱ) by the ethidium bromide model.

### Protein crystallization

All crystallization conditions were determined with crystal screens (Hampton) using sitting-drop vapor diffusion at 18 °C by mixing 0.5 μl samples and 0.5 μl reservoir solution. Crystals of the Se-Met NFIL3 (aa 65–161, 10 mg/ml) in apo form were obtained under conditions with 0.1 M Sodium cacodylate trihydrate pH 6.5 and 1.4 M Sodium acetate trihydrate. To obtain crystals of the NFIL3-DNA complex, the NFIL3 protein (aa 65–136, 6 mg/ml) was mixed with different DNA oligonucleotides at a molar ratio of 1:1.2, and both complex crystals were grown in a reservoir solution containing 0.1 M Sodium acetate trihydrate pH 5.6 and 8% w/v Polyethylene glycol 4000. The C/EBPα-DNA complex crystals were optimized and grown from drops mixed from 1.5 μl of protein complex solution (7 mg/ml) and 1.5 μl of reservoir solution (0.015 M Calcium chloride dihydrate, 0.1 M Sodium acetate trihydrate pH 4.6 and 30% v/v 2-Methyl-2,4-pentanediol). The C/EBPβ-DNA complex crystals were optimized and grown from drops mixed from 1.5 μl of protein complex solution (10 mg/ml) and 1.5 μl of reservoir solution (0.02 M Calcium chloride dihydrate, 0.1 M Sodium acetate trihydrate pH 4.6 and 20% v/v 2-Propanol).

### Data collection and structure determination

For data collection, crystals were cryoprotected in their reservoir solution supplemented with 25% v/v glycerol and flash-frozen in liquid nitrogen. Diffraction data of the NFIL3 in apo form were collected at SSRF 02U1 beamline at 100 K, while the diffraction data of the NFIL3-TTACGTAA DNA complex, NFIL3-TTATGTAA DNA complex, C/EBPα-TTACGTAA DNA complex, and C/EBPβ-TTACGTAA DNA complex were collected at SSRF 19U beamline at 100 K, respectively. All the data were then processed with the HKL 3000 suite (53), XDS (54), Phenix (55), and CCP4 (56). The Se-Met NFIL3 apo structures were determined using a single anomalous diffraction method with the AutoSol and AutoBuild programs embedded in the PHENIX suite (57, 58). The structures of the NFIL3-DNA complex were solved by the molecular replacement with the program PHASER (59) using the NFIL3 apo form as the search model. The C/EBPα-DNA and C/EBPβ-DNA structures were solved by the molecular replacement with the program PHASER using the structure of C/EBPα (PDB ID 1NWQ) and C/EBPβ (PDB ID 6MG1) as the search model, respectively. Model building was performed with Coot (60), and structure refinement was performed with REFMAC (Version 5.8.0257) (61). The crystal data collection and refinement statistics were summarized in Table S1. The structural models of NFIL3 bound to different DNA mutants were generated using Coot (60) based on the structure of the NFIL3-TTACGTAA complex, and presented by PyMol (62).

### Cell culture and Western blot

The HEK293T cells were maintained in Dulbecco’s modified Eagle’s medium containing 10% fetal bovine serum (Hyclone) and 0.1× Penicillin-Streptomycin (Hyclone) at 37 °C in 5% CO_2_ atmosphere. HEK293T cells cultured in 6-well plates (1 × 10^6^ cells/well) were transfected with 1 μg pCDNA3.1-NFIL3 (encoding Flag- and His_10_-tagged full-length NFIL3 wild-type or its mutants) with Lipofectamine 2000 (Invitrogen). Cells were transfected for 24 h, and were washed with 1× PBS twice. Cells were pelleted and lysed with lysis buffer on ice. Protein lysates were loaded on 10% gradient SDS-PAGE. After transferring to a PVDF membrane, the membrane was then blocked with 5% fat-free milk, and then incubated with 1 μl primary antibody probe (anti-Flag, Cat no: 66008-4-Ig, proteintech; anti-GAPDH, Cat no: 60004-1-Ig, proteintech) overnight at 4 °C. After washing the membrane with TBST three times, HRP-conjugated antibody (Cat no: SA00001-1, proteintech) was used for the secondary antibody probe. After washing by TBST, the membrane was incubated with 1 ml ECL reagent (Meilunbio) and exposed to Multi color fluorescent gel imaging system (FluorChem R).

### Luciferase activity analysis

The promoter sequence from −2k to −1 of the *IL-3* was predicted to contain one NFIL3 binding motif based on the JASPAR CORE database (https://jaspar.genereg.net/), which was confirmed by ITC assay and then subcloned into pGL 4.20 vector. The mutated pGL4.20 reporters were generated using mutated primers by PCR amplification, which was further confirmed by sequencing. The primer information of mutants was summarized in Table S2. HEK293T cultured in 24-well plates (2.5 × 10^5^ cells/well) were co-transfected with 400 ng pCDNA3.1-NFIL3, NFIL3 mutants and pCDNA3.1 (as control plasmid) respectively, 100 ng pGL4.20-*IL-3*-promoter, mutants and pGL4.20 (as control plasmid), respectively, and 5 ng Prl-TK (*Renilla* luciferase as an internal control) using Lipofectamine 2000 (Invitrogen). After transfection for 24 h, cells were washed with 1× PBS twice. Cells were pelleted and lysed with lysis buffer on ice. Luciferase activity was measured with the Dual-Luciferase reporter assay system (Promega) according to the manufacturer’s instructions using GloMax 20/20 Luminometer (Promega). Firefly luciferase activity was normalized based on the Renilla activity for each sample. Error bars represent SD from three replicates, which were calculated using one-way ANOVA by GraphPad Prism 7.

## Data availability

Coordinates and structure factors have been deposited in the Protein Data Base with accession numbers 8K89, 8K8A, 8K86, 8K8C, and 8K8D, respectively.

## Supporting information

This article contains supporting information.

## Conflict of interest

The authors declare no conflict of interest.
