# Supplementary material for: Structural basis for specific DNA sequence recognition by the transcription factor NFIL3
Source: J Biol Chem. 2024 Feb 19;300(3):105776. doi: 10.1016/j.jbc.2024.105776 (PMC10941009; doi:10.1016/j.jbc.2024.105776)
Supplement: Supporting information [file mmc1.pdf]

Supporting Information

Structural basis for specific DNA sequence motif recognition by the transcription factor NFIL3

Sizhuo Chen, Ming Lei, Ke Liu* and Jinrong Min*

Hubei Key Laboratory of Genetic Regulation and Integrative Biology, School of Life Sciences, Central China Normal University, Wuhan 430079, PR China

* To whom correspondence should be addressed. Email: minjinrong@ccnu.edu.cn or keliu2015@mail.ccnu.edu.cn.

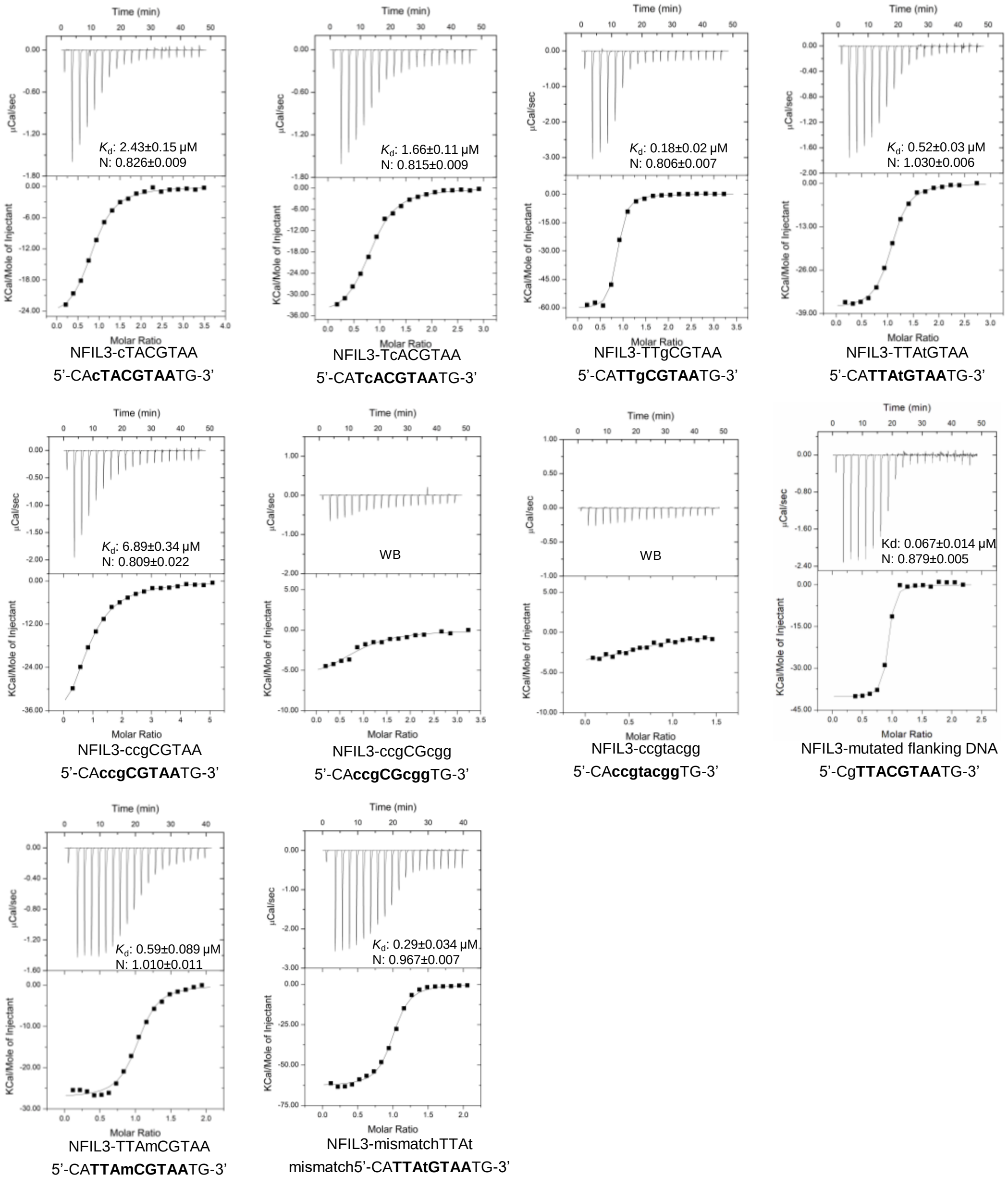

Figure S1. ITC binding curves of the NFIL3 bZIP domain to different DNA. One DNA strand sequence is shown. Protein recognition sequences are bolded. WB: weak binding.

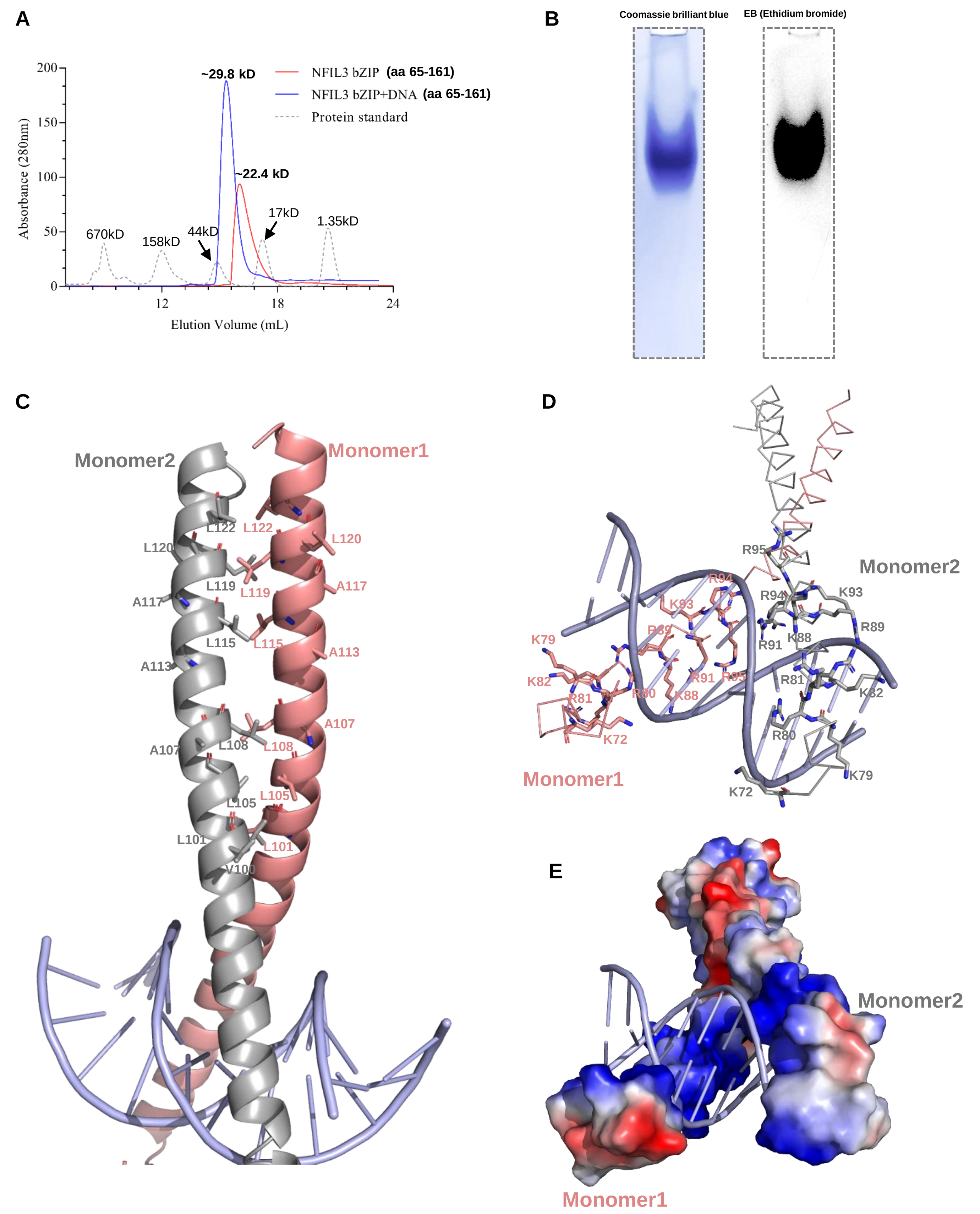

Figure S2. Overall structures of the bZIP domain of NFIL3 bound to DNA. (A) Size-exclusion chromatograms (SECs) of the NFIL3 bZIP domain alone and the NFIL3-DNA complex, respectively. The estimated molecular weights for the protein standards (Bio-rad), the bZIP dimer, and the bZIP-DNA complex of NFIL3 are labeled on their respective peaks. **(B)** Native-PAGE analysis of the SEC elution fractions corresponding to the NFIL3-DNA complex sample. The two identical samples were stained by Coomassie brilliant blue and EB, respectively. **(C)** Overall structure of the bZIP domain of NFIL3 in a cartoon representation. The hydrophobic residues are shown in stick models**. (D and E)** Complex structure of the basic region of the bZIP domain of NFIL3 in a cartoon representation **(D)** and electrostatic surface representation **(E)**, respectively. The positively charged residues are shown in stick models.

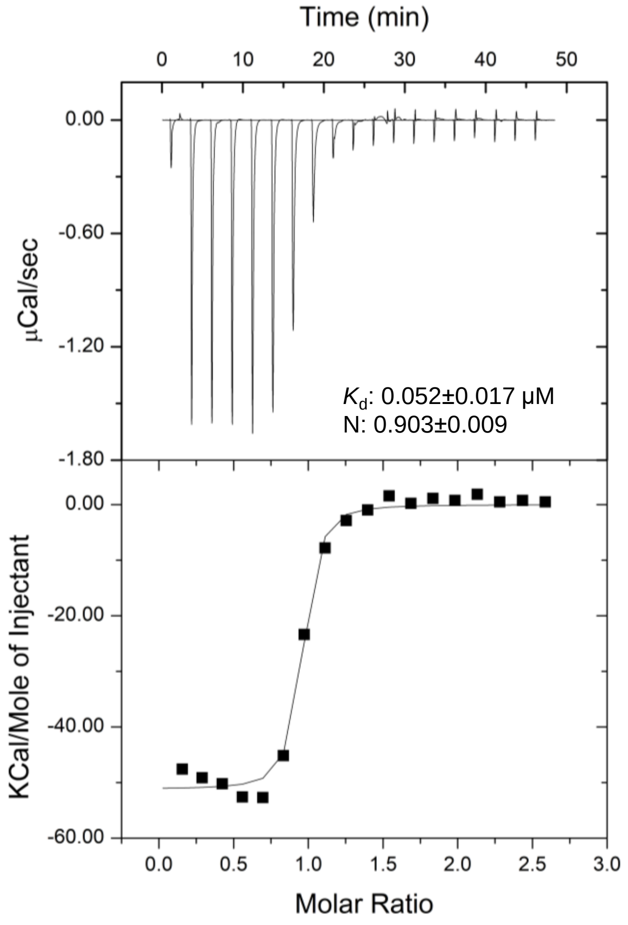

NFIL3 (65-136)-TTACGTAA

5'-CA**TTACGTAA**TG-3'

Figure S3. ITC binding curves of the short NFIL3 (aa 65-136) to TTACGTAA DNA. One DNA strand sequence is shown.

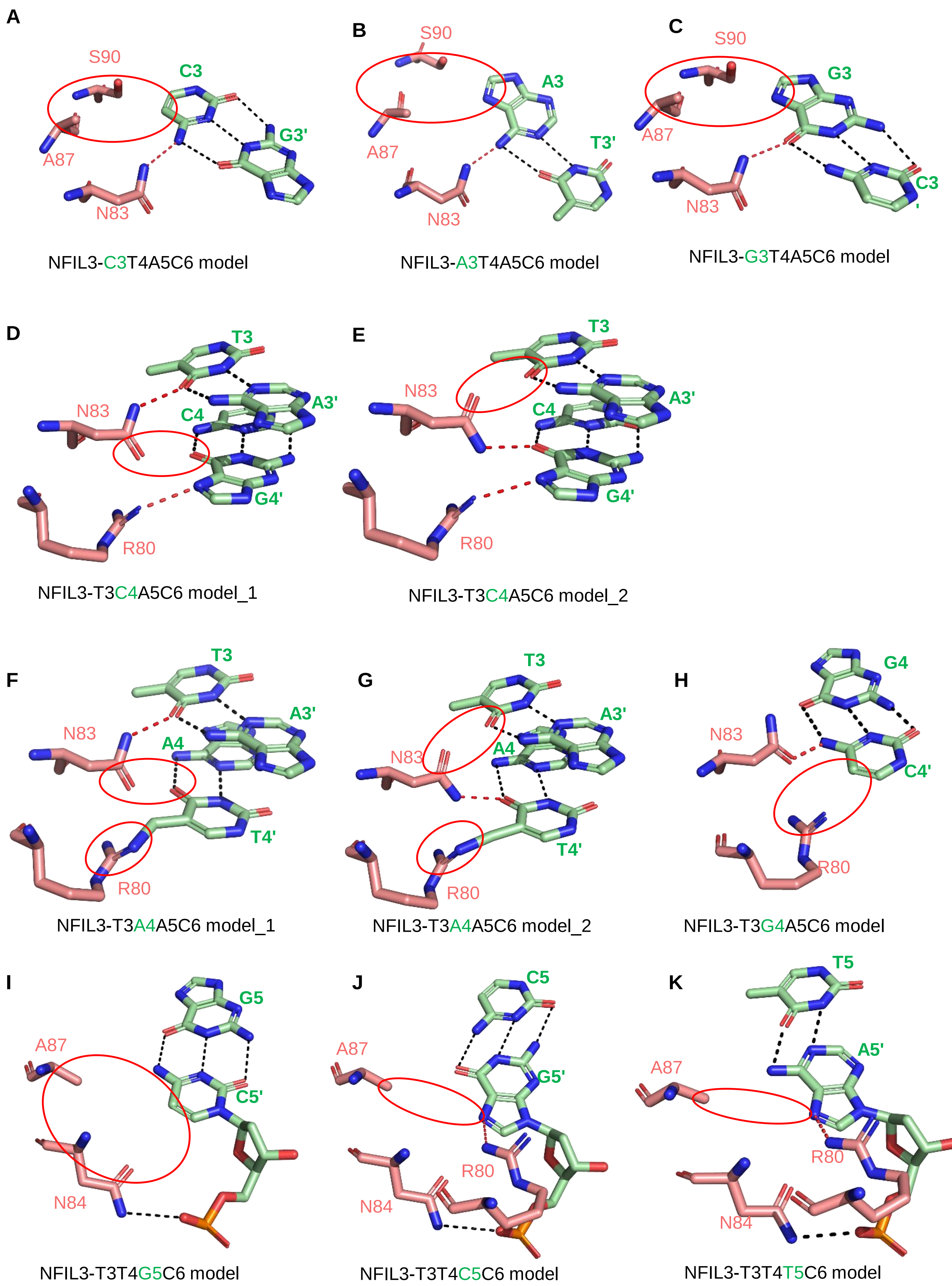

Figure S4. Structural models of NFIL3 bound to different DNA mutants. The protein residues and DNA bases are shown in stick models, and the mutated nucleotides are colored pale green. Hydrogen bonds formed between protein residues and bases, or between protein residues and backbone are marked as red and black dashed lines, respectively. The red rings represent the affected areas.

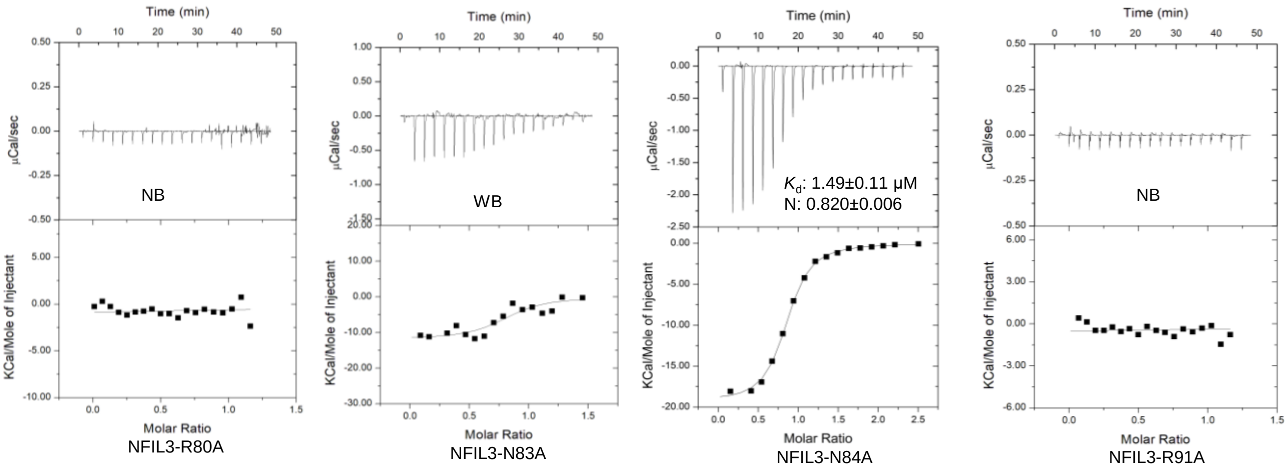

Figure S5. ITC binding curves of NFIL3 mutants to the TTACGTAA DNA. The DNA sequence of one strand of the DNA duplex is CATTACGTAATG. WB: weak binding. NB: no detectable binding.

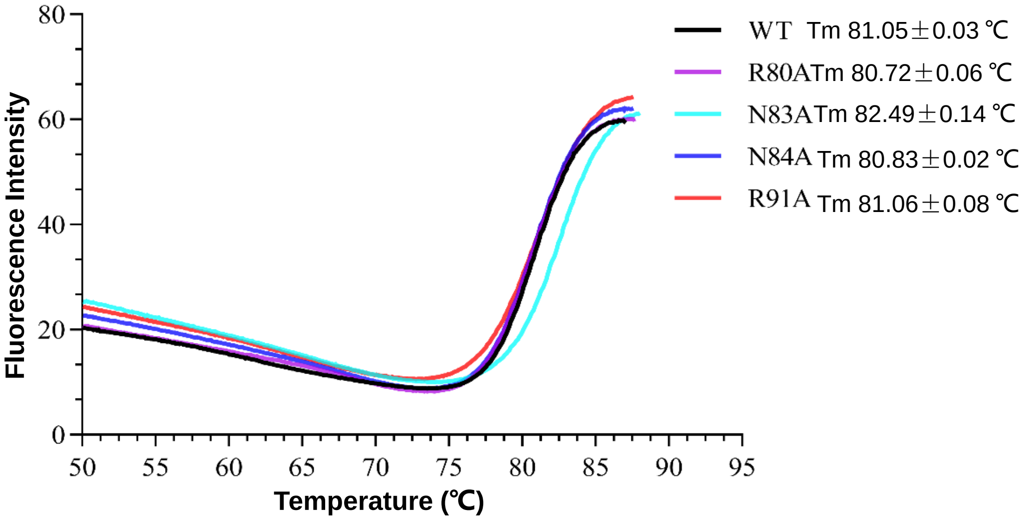

Figure S6. The protein stability analysis of the NFIL3 mutants by DSF analysis.

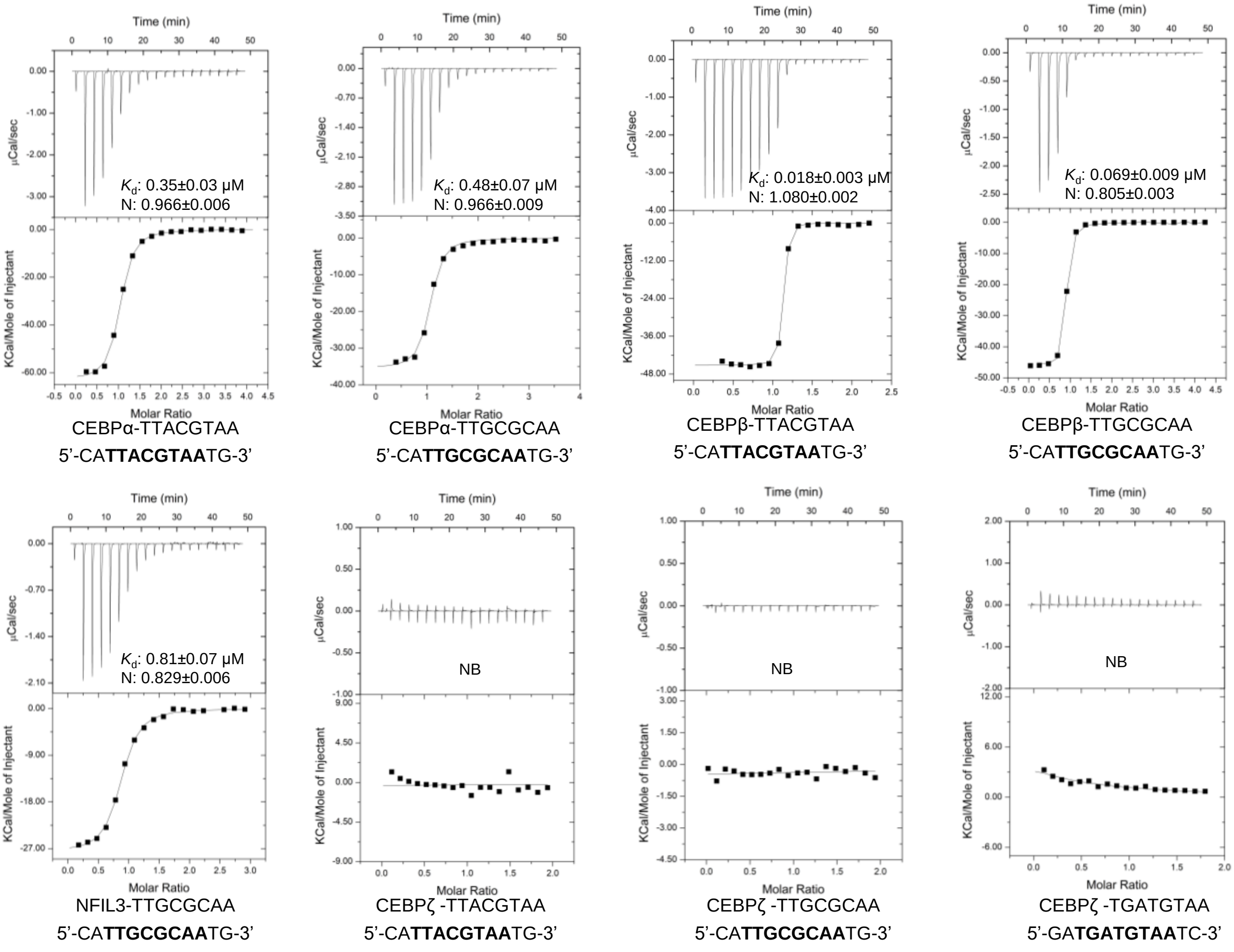

Figure S7. ITC binding curves of the bZIP domains of NFIL3, C/EBPα, C/EBPβ and C/EBPζ to different DNA mutants. One DNA strand sequence is shown. NB: no detectable binding.

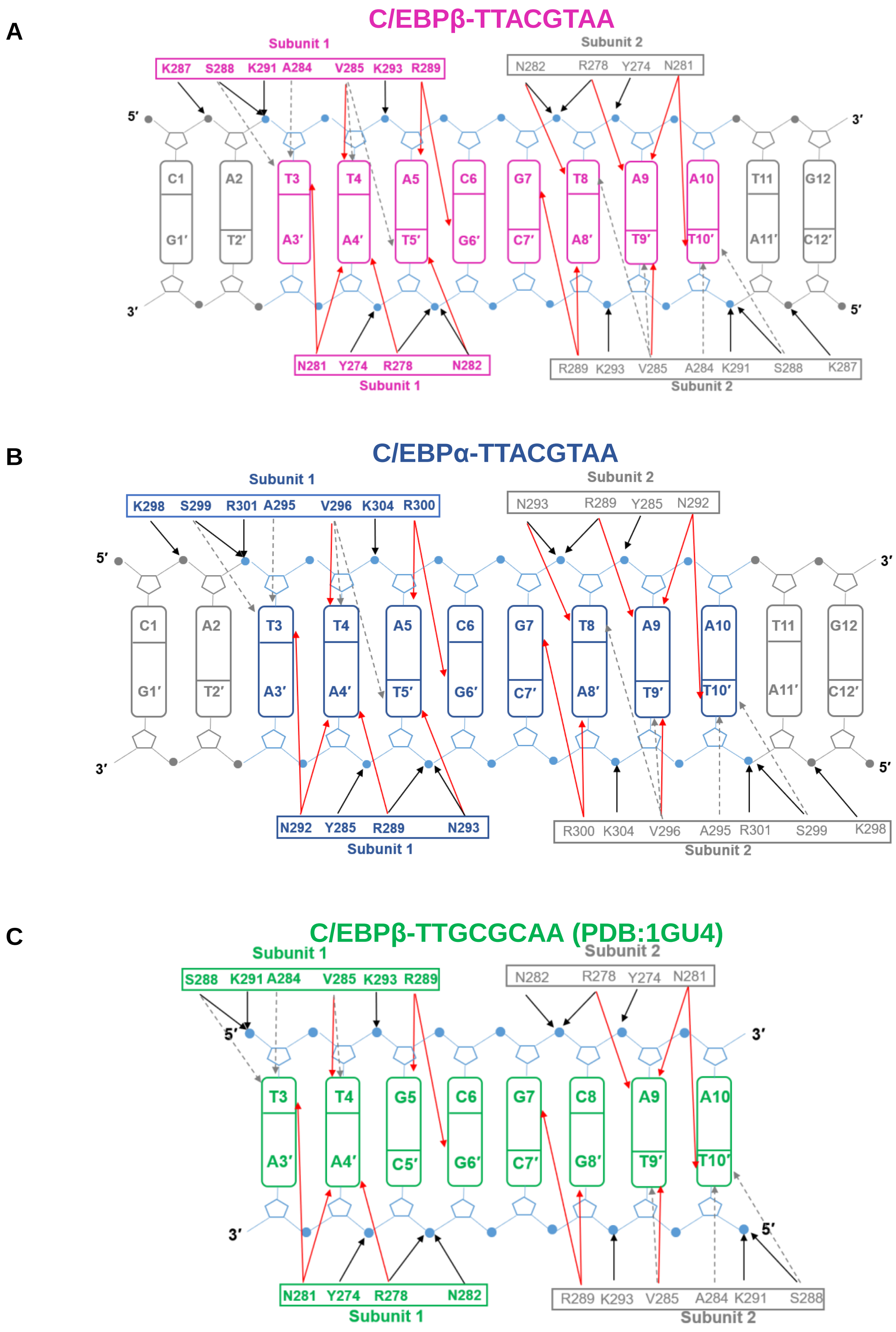

Figure S8. Schematic diagrams of the bZIP domains of C/EBPα and C/EBPβ bound to the TTAC and TTGC DNA. Schematic diagrams of C/EBPβ bound to TTACGTAA DNA **(A)**, C/EBPα bound to TTACGTAA DNA **(B)**, and C/EBPβ bound to TTGCGCAA DNA PDB 1GU4 **(C)**, respectively. Hydrogen bonds of DNA base-specific and backbone interactions are represented as red and black solid arrows, respectively. Van der Waals interactions between the residues and DNA are represented as gray dotted arrows.

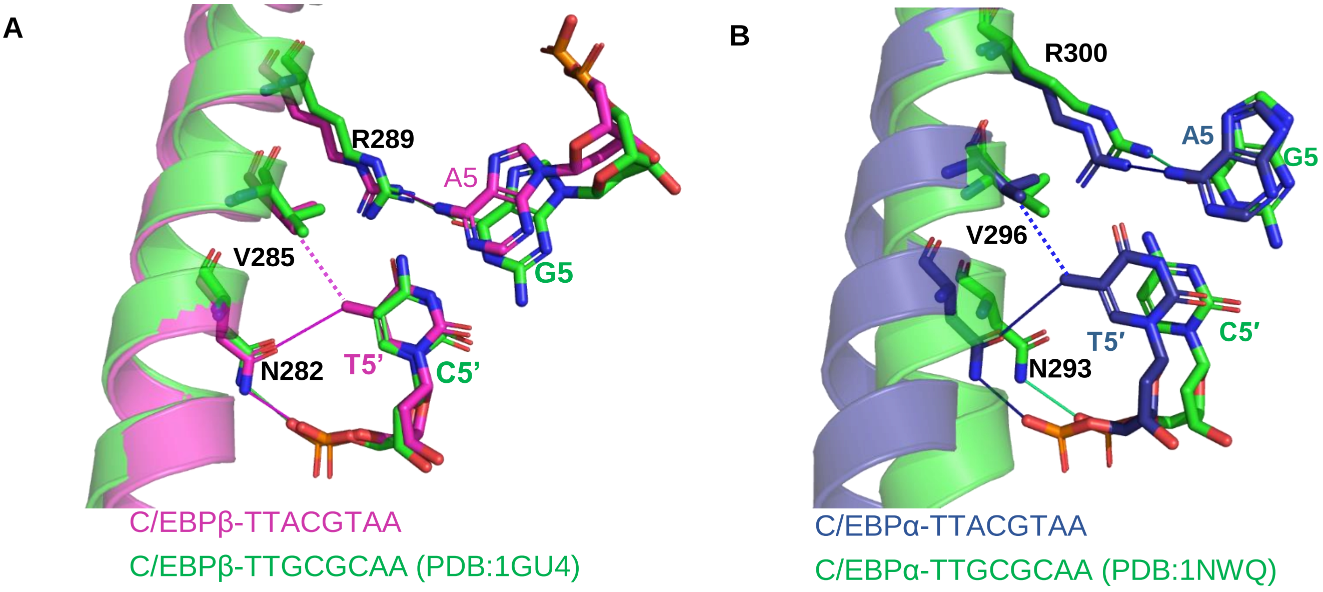

Figure S9. Closed view of the bZIP domains of C/EBPα and β bound to DNA. (A) The interaction difference between C/EBPβ with A5/T5’ base pair and G5/C5’ base pair. **(B)** The interaction difference between C/EBPα with A5/T5’ base pair and G5/C5’ base pair. The base interacting residues and DNA bases are shown in sticks. Hydrogen bonds and Van der Waals interactions are represented as solid and dotted lines, respectively.

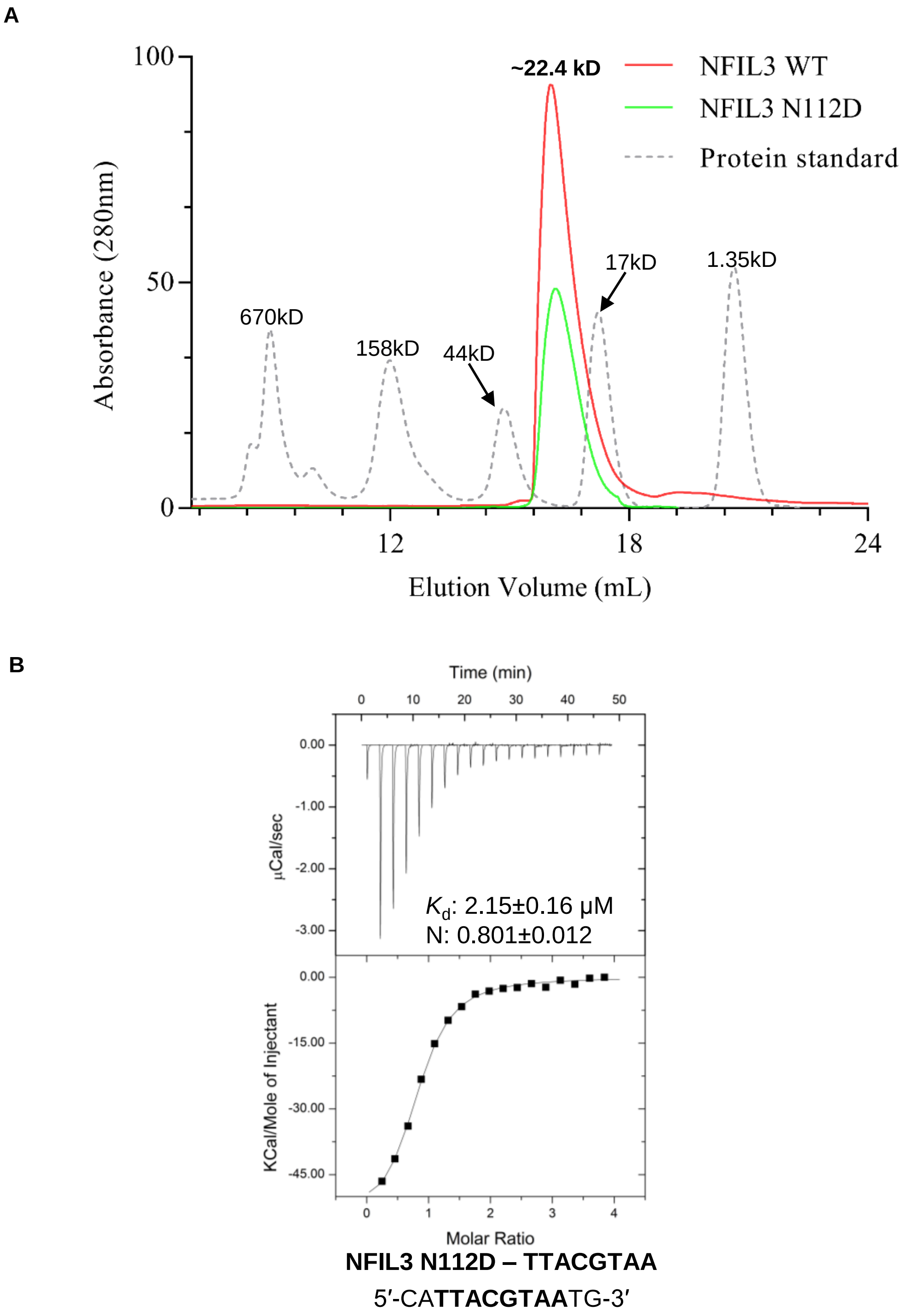

Figure S10. DNA binding ability and size-exclusion chromatograms (SECs) of the NFIL3 N112D mutant. **(A)** The SEC analysis of the the bZIP WT and N112D mutant. **(B)** ITC binding curve of NFIL3 N112D mutant to the TTACGTAA DNA.

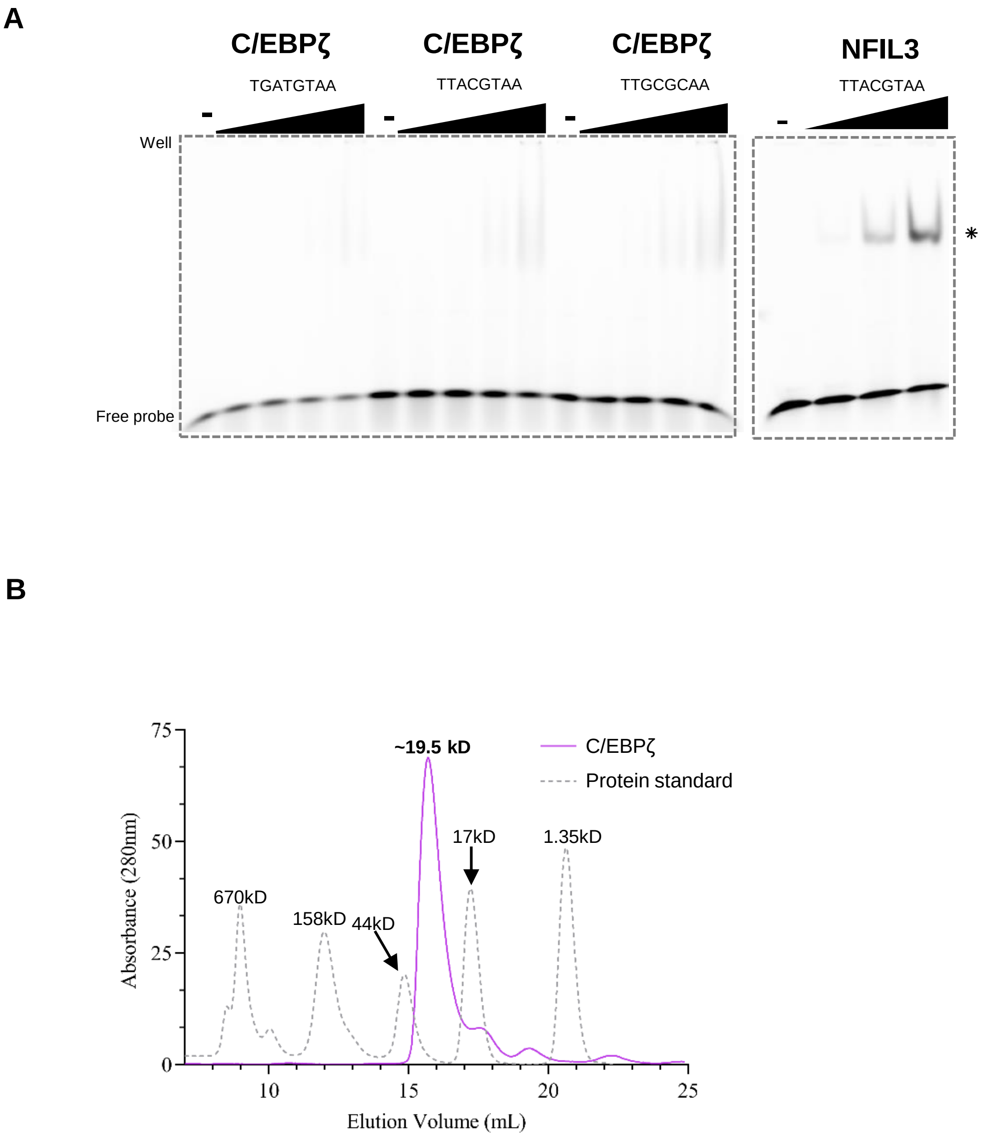

Figure S11. Verification and analysis of CEBPζ DNA-binding affinity. (A) EMSA assays of the C/EBPζ bZIP domain bound to different DNA. The final concentrations of proteins in each set are 6, 12, 24 and 48 μM, respectively. NFIL3 bZIP bound to TTACGTAA as positive control, and the final concentrations of proteins in each set are 6, 12, 24. - : only DNA control. ⁎ represent bound DNA bands. The gels were uniformly exposed to 10s. **(B)** Size-exclusion chromatograms (SECs) of the C/EBPζ bZIP domain. The molecular weights for the protein standards (Bio-rad) and the bZIP dimer of C/EBPζ are labeled for each peak.

Mutations	Project	Disease Type
**E78G**	TCGA-STAD	➢ Adenomas and Adenocarcinomas<br>➢ Cystic, Mucinous and Serous Neoplasms
**R91H**	TCGA-COAD<br>TCGA-UCEC	➢ Adenomas and Adenocarcinomas<br>➢ Cystic, Mucinous and Serous Neoplasms<br>➢ Epithelial Neoplasms, NOS<br>➢ Complex Epithelial Neoplasms
**R91C**	TCGA-UCEC	➢ Adenomas and Adenocarcinomas<br>➢ Cystic, Mucinous and Serous Neoplasms<br>➢ Epithelial Neoplasms, NOS
**R94H**	TCGA-UCEC	➢ Adenomas and Adenocarcinomas<br>➢ Cystic, Mucinous and Serous Neoplasms<br>➢ Epithelial Neoplasms, NOS
**R95Q**	TCGA-UCEC	➢ Adenomas and Adenocarcinomas<br>➢ Cystic, Mucinous and Serous Neoplasms<br>➢ Epithelial Neoplasms, NOS
**E111Q**	MMRF-COMMPASS	➢ Plasma Cell Tumors
**A113T**	TCGA-UCEC<br>CPTAC-3	➢ Adenomas and Adenocarcinomas<br>➢ Cystic, Mucinous and Serous Neoplasms<br>➢ Epithelial Neoplasms, NOS<br>➢ Ductal and Lobular Neoplasms<br>➢ Gliomas<br>➢ Squamous Cell Neoplasms
**A113V**	TCGA-UCEC	➢ Adenomas and Adenocarcinomas<br>➢ Cystic, Mucinous and Serous Neoplasms<br>➢ Epithelial Neoplasms, NOS

Figure S12. The NFIL3 bZIP domain mutants associated diseases in The Cancer Genome Atlas (TCGA) database.

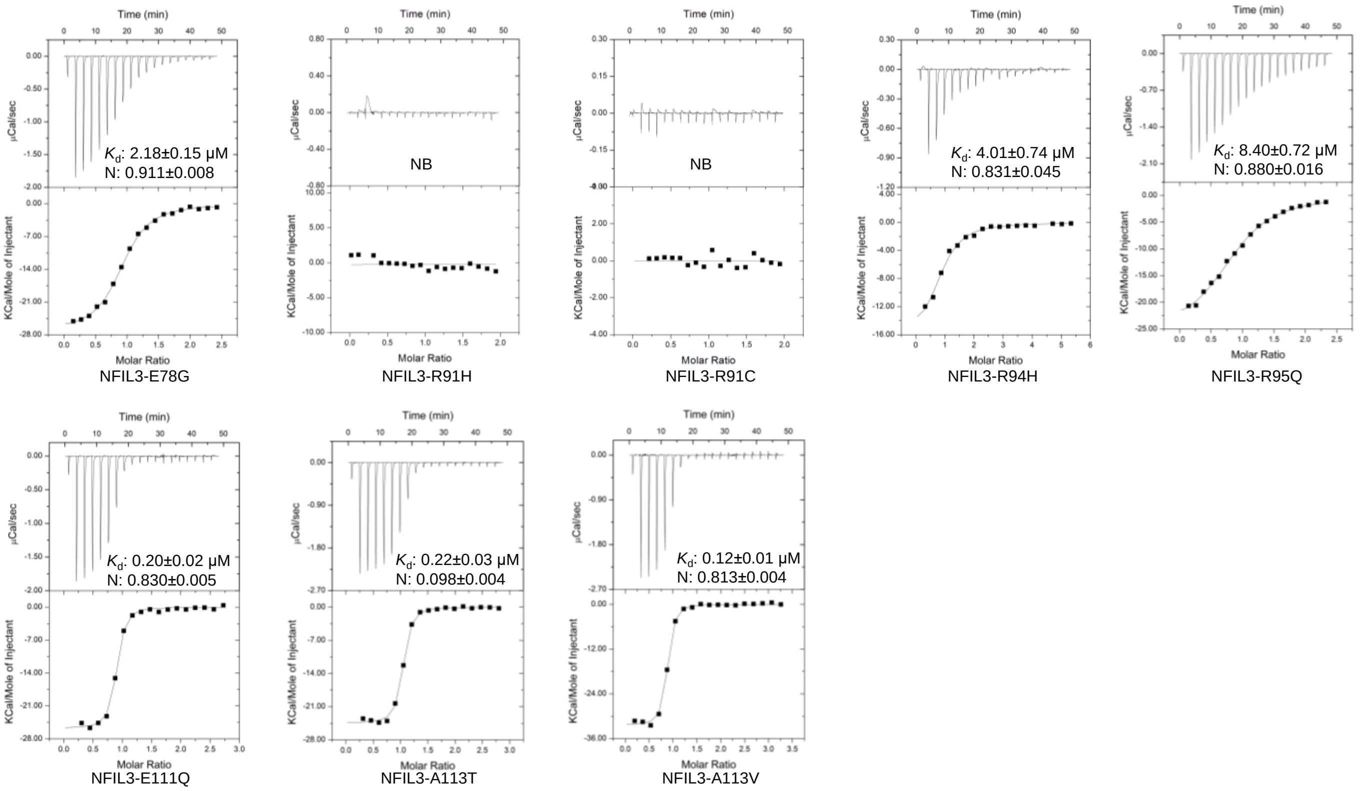

Figure S13. ITC binding curves of NFIL3 disease-associated mutants to the TTACGTAA DNA. One DNA strand sequence is CATTACGTAATG. NB: no detectable binding.

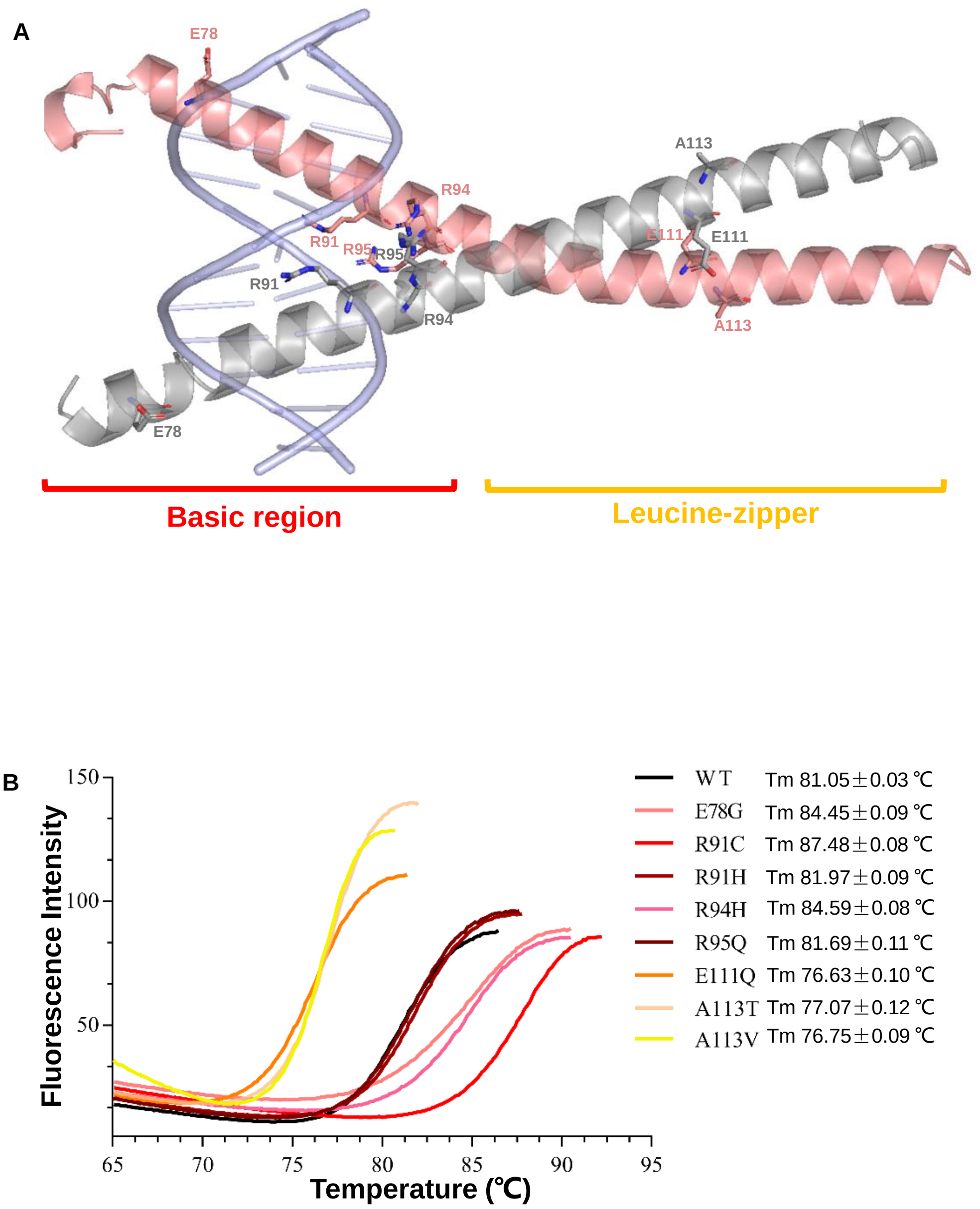

Figure S14. The protein stability analysis of NFIL3 disease-relevant mutants. (A) Disease-causing mutations in the bZIP domains of NFIL3. The disease-causing mutations residues are shown in stick models. **(B)** The DSF analysis for the NFIL3 mutants. Mutants in basic region are indicated by the red line, mutants in leucine zipper region are indicated by the yellow line.

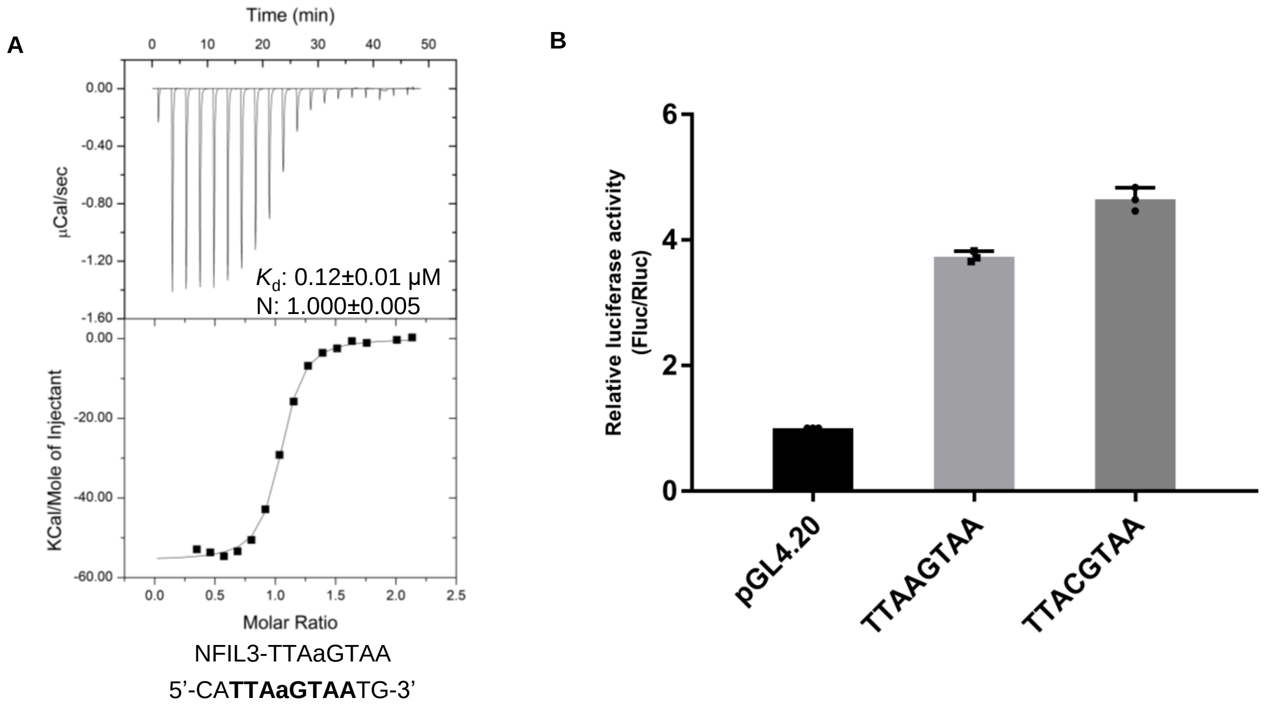

Figure S15. ITC binding curve and Dual luciferase assays of NFIL3 to the TTAAGTAA and TTACGTAA DNA. (A) ITC binding curve of NFIL3 to TTAAGTAA DNA. One DNA strand sequence is CATTACGTAATG. (B) Dual luciferase assays of NFIL3 binding to WT and TTACGTAA *IL-3*-promoters in HEK293T. pGL4.20: empty vector as a negative control. Error bars represent SD from three replicates (n = 3) (**P < 0.01).

Supplementary Table S1. Data collection and refinement statistics

**Structure**	**SeMET-NFIL3 (aa 65–161)**	**NFIL3-TTACGTAA (aa 65–136)**	**NFIL3-TTATGTAA (aa 65–136)**	**C/EBPα-TTACGTAA (aa 281–340)**	**C/EBPβ-TTACGTAA (aa 259–336)**
**DNA sequence**		**5’-CATTACGTAATG-3’ 3’-GTAATGCATTAC-5’**	**5’-CATTATGTAACG-3’ 3’-GTAATACATTGC-5’**	**5’-CATTACGTAATG-3’ 3’-GTAATGCATTAC-5’**	**5’-CATTACGTAATG-3’ 3’-GTAATGCATTAC-5’**
**PDB ID**	**8K89**	**8K8A**	**8K86**	**8K8C**	**8K8D**
**Data Collection**					
Space group	$P2_12_12_1$	$P2_12_12_1$	$P2_12_12_1$	$P2_12_12_1$	C2
**Cell dimensions**					
a,b,c [Å]	40.12, 65.68, 85.22	74.03, 96.95, 38.95	74.08, 97.52, 39.01	39.38 63.37 128.55	63.67 46.40 98.78
α, β, γ [°]	90, 90, 90	90, 90, 90	90, 90, 90	90, 90, 90	90, 108.76, 90
Resolution [Å]	35.75-2.10 (2.08-1.97) [a]	58.82-2.06 (2.17-2.06)	58.99-2.06 (2.18-2.06)	64.27-2.06 (2.17-2.06)	32.14-2.20 (2.27-2.20)
Completeness [%]	91.0 (99.9)	98.8 (99.9)	100 (100)	99.9 (100)	99.9 (99.7)
R_{merge}[b]	0.061 (0.578)	0.055 (0.522)	0.091 (0.668)	0.068 (0.947)	0.034 (0.231)
I/σI	13.7 (2.9)	16.7 (2.6)	14.7 (2.7)	18.9 (2.7)	15.6 (2.7)
CC1/2	0.997 (0.749)	0.999 (0.904)	0.998 (0.916)	1.000 (0.938)	0.996 (0.898)
Redundancy	5.5 (4.7)	6.9 (5.6)	9.8 (8.6)	12.5 (12.8)	1.9 (1.9)
**Refinement**					
Resolution (Å)	19.52-2.10	28.09-2.07	19.91-2.06	19.96-2.06	30.13-2.20
Reflections (working set)	12493	17534	17941	20673	13944
Reflections (test set)	609	921	1794	1986	701
No. atoms/B-factor ($Å^2$)	1238/69.42	1557/48.55	1623/40.88	1598/51.57	1669/57.66
Protein	610/69.53	498/47.76	499/39.29	508/54.26	571/60.67
DNA		243/50.67	264/44.17	263/46.76	243/50.94
Water	17/54.44	74/46.07	138/41.49	55/48.89	58/54.24
R_{work}/R_{free}[c]	0.235/0.288	0.227/0.276	0.223/0.259	0.225/0.281	0.236/0.291
RMS deviations					
Bond lengths (Å)	0.010	0.010	0.010	0.010	0.010
Bond angles (°)	0.97	0.94	0.88	1.07	1.03
**Ramachandran Plot % residues**					
Favoured	97.9%	99.2%	99.1%	100%	97.7%
Allowed	2.1%	0.8%	0.9%	0%	2.3%
Outliers	0	0	0	0	0

[a] Numbers in parentheses represent the highest resolution shell.

[b] Rmerge = ∑hkl∑i|Ii(hkl)−<I(hkl)>|/∑hkl∑iIi(hkl).

[c] R-factor = ∑hkl||Fo|−|Fc||/∑hkl|Fo|.

Supplementary Table S2. The primer information

Primers	Sequence (5'-3')
NFIL3-pcDNA1F	TTGTATTTCCAGGGCATGATGCAGCTGAGAAAAATGCAGACCGTC
NFIL3-pcDNA462R	CCACACTGGACTAGTTTATTACCCAGAGTCTGAAGCAGAGATTGG
NFIL3-pcE78GF	AAGAAAGATGCTATGTATTGGGGTAAAAGGCGGAAAAATAATGAA
NFIL3-pcE78GR	AGCTTCATTATTTTTCCGCCTTTTACCCCAATACATAGCATCTTTCTT
NFIL3-pcR91CF	AATGAAGCTGCCAAAAGATCTCATGAGAAGCGTCGACTGAATGAC
NFIL3-pcR91CR	CAGGTCATTCAGTCGACGCTTCTCATGAGATCTTTTGGCAGCTTC
NFIL3-pcR91HF	AATGAAGCTGCCAAAAGATCTTGTGAGAAGCGTCGACTGAATGAC
NFIL3-pcR91HR	CAGGTCATTCAGTCGACGCTTCTCACAAGATCTTTTGGCAGCTTC
NFIL3-pcR94HF	GCCAAAAGATCTCGTGAGAAGCATCGACTGAATGACCTGGTTTTA
NFIL3-pcR94HR	TAAAACCAGGTCATTCAGTCGATGCTTCTCACGAGATCTTTTGGC
NFIL3-pcR95QF	AAAAGATCTCGTGAGAAGCGTCAGCTGAATGACCTGGTTTTAGAG
NFIL3-pcR95QR	CTCTAAAACCAGGTCATTCAGCTGACGCTTCTCACGAGATCTTTT
IL-3promoter-2000F	CTGGCCTCGGCGGCCTGTGCACAAGTCCCCAAAACGTTT
IL-3promoter-0R	CAGTACCGGATTGCCTGAAGAGTTGGCAACAGCCTCCCG
IL-3promoter-mut0F	GACAAGATGAAGTGATACCGTTCTTTTTTCTTGTTTCACTGA
IL-3promoter-mut0R	TCAGTGAAACAAGAAAAAAGAACGGTATCACTTCATCTTGTC
IL-3promoter-mut1F	GACAAGATGAAGTGATACCGTTTAGTAATCTTTTTTCTTGTTTCAC
IL-3promoter-mut1R	TCAGTGAAACAAGAAAAAAGATTACTAAACGGTATCACTTCATCTT
IL-3promoter-mut2F	GACAAGATGAAGTGATACCGTTTAACGTAATCTTTTTTCTTGTTT
IL-3promoter-mut2R	TCAGTGAAACAAGAAAAAAGATTACGTTAAACGGTATCACTTCAT
NFIL3-65F	TTGTATTTCCAGGGCGAGTTCATACCGGATGAAAAAAAG
NFIL3-E111DF	TTAATTGCATTGGGTGAAGACAATGCGACCTTGAAAGCCGAGCTG
NFIL3-E111DR	CAGCTCGGCTTTCAAGGTCGCATTGTCTTCACCCAATGCAATTAA
NFIL3-136R	CTCGAGTGCGGCCTCATCATTGAGCATAGGCGGTTGAGGAAATTAA
NFIL3-161R	CTCGAGTGCGGCCTCATCAAGAACTAACGTTAGACTTTGAAGT
NFIL3-R80AF	GATGCTATGTACTGGGAGAAGGCACGCAAAAATAACGAAGCAGCG
NFIL3-R80AR	CGCTGCTTCGTTATTTTTGCGTGCCTTCTCCCAGTACATAGCATC
NFIL3-N83AF	TACTGGGAGAAGCGCCGCAAAGCAAACGAAGCAGCGAAACGG
NFIL3-N83AR	CGACCGTTTCGCTGCTTCGTTTGCTTTGCGGCGCTTCTCCCAGTA
NFIL3-N84AF	TGGGAGAAGCGCCGCAAAAATGCAGAAGCAGCGAAACGGTCG
NFIL3-N84AR	ACGCGACCGTTTCGCTGCTTCTGCATTTTTGCGGCGCTTCTCCCA
NFIL3-R91AF	AACGAAGCAGCGAAACGGTCGGCAGAAAAGAGAAGATTGAAT
NFIL3-R91AR	GTCATTCAATCTTCTCTTTTCTGCCGACCGTTTCGCTGCTTCGTT
NFIL3-N112DF	TTAATTGCATTGGGTGAAGAGGATGCGACCTTGAAAGCCGAGCTG
NFIL3-N112DR	CAGCTCGGCTTTCAAGGTCGCATCCTCTTCACCCAATGCAATTAA
NFIL3-E78GF	AAAAAGGATGCTATGTACTGGGGTAAGCGCCGCAAAAATAACGAA
NFIL3-E78GR	TTCGTTATTTTTGCGGCGCTTACCCCAGTACATAGCATCCTTTTT
NFIL3-R91HF	AATAACGAAGCAGCGAAACGGTCGCATGAAAAGAGAAGATTG
NFIL3-R91HR	ATTCAATCTTCTCTTTTCATGCGACCGTTTCGCTGCTTCGTTATTTTT
NFIL3-R91CF	AATAACGAAGCAGCGAAACGGTCGTGTGAAAAGAGAAGATTG
NFIL3-R91CR	ATTCAATCTTCTCTTTTCACACGACCGTTTCGCTGCTTCGTTATT
NFIL3-R94HF	GCGAAACGGTCGCGTGAAAAGCATAGATTGAATGACCTTGTCTTA

NFIL3-R94HR	TAAGACAAGGTCATTCAATCTATGCTTTTCACGCGACCGTTTCGC
NFIL3-R95QF	AAACGGTCGCGTGAAAAGAGACAGTTGAATGACCTTGTCTTAGAG
NFIL3-R95QR	CTCTAAGACAAGGTCATTCAACTGTCTCTTTTCACGCGACCGTTT
NFIL3-E111QF	AACAAGTTAATTGCATTGGGTGAACAGAATGCGACCTTGAAAGCC
NFIL3-E111QR	CTCGGCTTTCAAGGTCGCATTCTGTTCACCCAATGCAATTAACTT
NFIL3-A113TF	ATTGCATTGGGTGAAGAGAATACCACCTTGAAAGCCGAGCTGTTG
NFIL3-A113TR	CAACAGCTCGGCTTTCAAGGTGGTATTCTCTTCACCCAATGCAAT
NFIL3-A113VF	ATTGCATTGGGTGAAGAGAATGTTACCTTGAAAGCCGAGCTGTTG
NFIL3-A113VR	CAACAGCTCGGCTTTCAAGGTAACATTCTCTTCACCCAATGCAATT
C/EBPα-281F	TTGTATTTCCAGGGCAACAGTAATGAGTACAGAGTGAGAAGA
C/EBPα-D320EF	GTGCTGGAGCTGACAAGCGAAAACGAAAGACTGAGAAAGAGA
C/EBPα-D320ER	CACTCTCTTTCTCAGTCTTTCGTTTTCGCTTGTCAGCTCCAGCAC
C/EBPα-340R	CTCGAGTGCGGCCTCATCATCCTCTCAGTGTGTCCAGCTCTCT
C/EBPβ-259F	TTGTATTTCCAGGGCGTCAAGAGCAAGGCCAAGAAGACC
C/EBPβ-E309DF	AAGGTCCTGGAGCTCACGGCCGACAACGAGCGGCTGCAGAAG
C/EBPβ-E309DR	CTTCTTCTGCAGCCGCTCGTTGTCGGCCGTGAGCTCCAGGACCTT
C/EBPβ-336R	CTCGAGTGCGGCCTCATCACTCGGGCAGCTGCTTGAACAAGTT
C/EBPζ-88F-W	TTGTATTTCCAGGGCAGCTCCCTGGCTCAGGAGGAAGAGGAG
C/EBPζ-162R	CTCGAGTGCGGCCTCATCATGCTTGGTGCAGATTCACCATTCGGTC
